# Whole microbiota transplantation restores gut homeostasis throughout the gastrointestinal tract

**DOI:** 10.1002/imt2.70091

**Published:** 2025-11-11

**Authors:** Bufu Tang, Yuan Cao, Jiasu Li, Nan Gao, Pingting Gao, Xiaochao Chen, Zunzhen Ming, Zhaoshen Li, Weiliang Hou

**Affiliations:** ^1^ Department of Interventional Radiology, Zhongshan Hospital, Shanghai Institute of Medical Imaging, National Clinical Research Center of Interventional Medicine Fudan University Shanghai China; ^2^ Institute of Clinical Science, Zhongshan Hospital Fudan University Shanghai China; ^3^ Department of Gastroenterology, Shanghai Institute of Pancreatic Diseases, National Key Laboratory of Immunity and Inflammation, Changhai Clinical Research Unit, Changhai Hospital Naval Medical University Shanghai China; ^4^ Shanghai Collaborative Innovation Center of Endoscopy, Endoscopy Center and Endoscopy Research Institute of Zhongshan Hospital Fudan University Shanghai China; ^5^ Department of Proctology Chengdu Anorectal Hospital Chengdu China; ^6^ School of Life Sciences Shanghai University Shanghai China

## Abstract

This study introduces whole microbiota transplantation (WMT), a synergistic therapeutic approach that concurrently transplants small intestinal and fecal microbiota. In germ‐free mice, WMT outperforms conventional fecal microbiota transplantation (FMT) in restoring gut microbiota diversity and abundance. Moreover, in a chemotherapy‐induced intestinal mucositis model, WMT alleviates intestinal inflammation and reverses microbiota dysbiosis. Encapsulation in layer‐by‐layer self‐assembled nanocapsules further boosts microbial survival and colonization, amplifying WMT's anti‐inflammatory effects and microbiota restoration in a mouse model of pan‐intestinal infection. Overall, WMT represents a precise strategy for reshaping microbial homeostasis across the entire gastrointestinal tract, with therapeutic promise for inflammatory bowel diseases and small‐intestinal disorders.

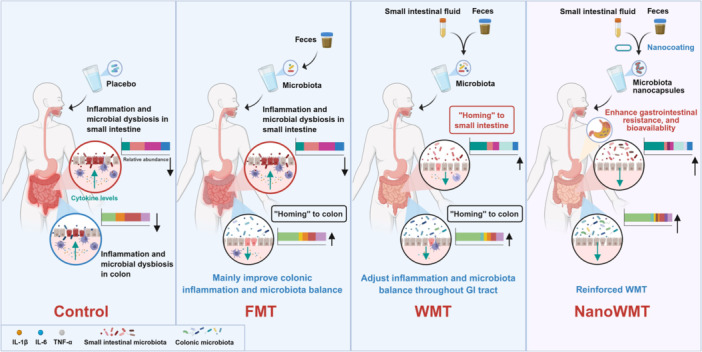

To the Editor,

The gut microbiota comprises distinct microbial communities across the small intestine, cecum, and colon that dynamically interact with the host to maintain intestinal homeostasis [1]. Fecal microbiota transplantation (FMT) has emerged as a promising therapeutic modality for restoring microbial and metabolic balance in various diseases, particularly recurrent *Clostridioides difficile* infection and inflammatory bowel diseases [2]. However, due to the spatial heterogeneity and “homing” properties of microbes along the gastrointestinal (GI) tract [3], FMT primarily modulates colonic microbiota without substantially altering small intestinal bacterial communities [4]. Consequently, FMT exhibits limited efficacy in small intestine‐related disorders, including small intestinal bacterial overgrowth, irritable bowel syndrome, and short‐bowel syndrome [5].

The small intestine and its microbiota play critical roles in nutrient absorption, host metabolism, and immune modulation [6]. Unlike colonic or fecal microbiota, the small intestinal microbiota (SIM) harbors a higher proportion of facultative anaerobic and aerobic bacteria, such as *Lactobacillus*, *Clostridium*, *Staphylococcus*, and *Streptococcus*, reflecting distinct luminal conditions, including pH gradients and oxygen concentration [5, 6]. Recently, SIM transplantation was shown to favorably modulate mucosal ecosystems and host metabolic pathways in both murine and human studies [4], and clinical protocols for SIM transplantation have been established [7].

Here, we developed a whole microbiota transplantation (WMT) strategy combining SIM and fecal microbiota to comprehensively treat intestinal inflammation. In germ‐free (GF) mice, WMT showed superior efficacy to FMT in restoring microbiota diversity and composition across the small intestine, cecum, and colon, enriching beneficial taxa while suppressing potential pathogens. In a chemotherapy‐induced intestinal mucositis model, WMT significantly ameliorated intestinal shortening, attenuated inflammatory responses, and corrected dysbiosis. To enhance tolerance to GI conditions, we engineered a chitosan‐alginate nanoshell for microbiota delivery. In a *Salmonella typhimurium* (STm) infection model, nanocapsule‐assisted WMT (NanoWMT) further augmented the anti‐inflammatory effects and microbiota restoration achieved by WMT alone. Collectively, WMT represents a more precise therapeutic strategy than FMT, enabling simultaneous regulation of microbial homeostasis throughout the GI tract with promising clinical applicability.

## WMT RESTORES GUT MICROBIOTA OF GF MICE

To establish a novel microbiota transplantation approach, we first evaluated WMT in GF mice. GF mice were orally administered with the equal doses of FMT or WMT. After 24 h, mice were euthanized, and contents from the small intestine, cecum, and colon were collected for 16S rRNA sequencing. In the small intestine, gut microbiota alpha and beta diversity of the microbiota were higher in WMT‐treated mice than in FMT‐treated mice (Figures S1A,B, S2). The relative abundance of the gut microbiota shifted significantly at both the phylum and genus levels in the WMT group (Figure S1C). Compared to the FMT group, Firmicutes abundance increased markedly in the WMT group (Figure S1D). Notably, WMT increased *Turicibacter* levels, which contributes to host bile acid and lipid metabolism [8], while reducing *Enterococcus*, a potential pro‐inflammatory pathogen (Figure S1D) [9].

WMT also substantially increased microbiota diversity in the cecum and colon, as evidenced by elevated Shannon indices and principal component analysis of beta diversity (Figure S1E,F,I,J). Taxa relative abundance shifted at both the phylum and genus levels in the WMT group (Figure S1G,K). In the cecum, Firmicutes and beneficial genera such as *Akkermansia*, *Faecalibaculum*, and *Lachnospiraceae_NK4A136_group* increased significantly, supporting intestinal barrier integrity (Figure S1H) [10, 11]. In the colon, the Firmicutes*/*Proteobacteria ratio and *Turicibacter* abundance increased, whereas Proteobacteria and *Escherichia‐Shigella* decreased (Figure S1L). Linear discriminant analysis effect size (LEfSe) analysis identified 30 differentially abundant species between the FMT and WMT groups across all three intestinal segments (Figure S2). Microbiota function prediction indicated that WMT significantly upregulated multiple metabolic pathways in the colon of GF mice, including those for carbohydrate, lipid, and amino acid metabolism (Figure S3). These findings indicate that WMT surpasses FMT in restoring the structure and function of the gut microbiota.

## WMT AMELIORATES INTESTINAL MUCOSITIS AND RESHAPES MICROBIOTA DYSBIOSIS

**Figure 1:**
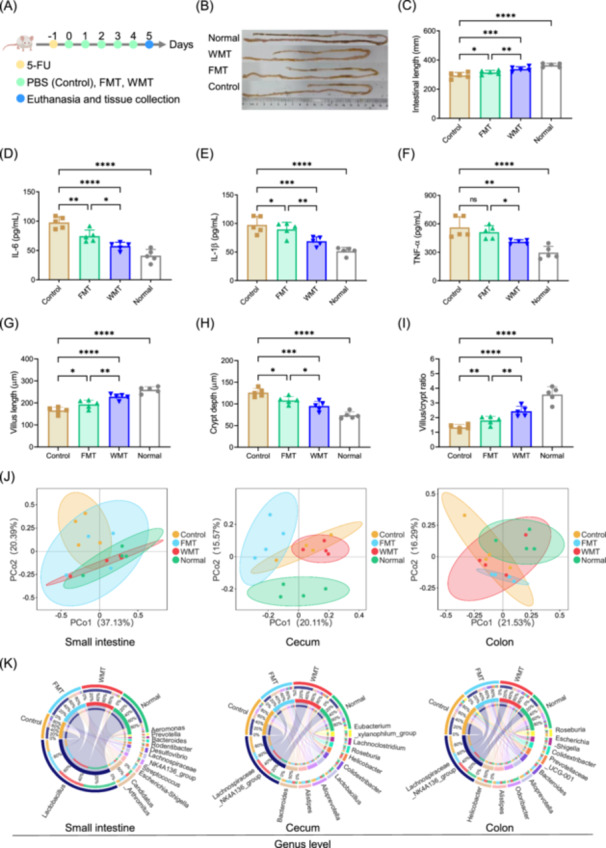
WMT ameliorates 5‐fluorouracil (5‐FU)‐induced intestinal mucositis. (A) Experimental timeline for the 5‐FU‐induced mucositis model (*n* = 5 per group). (B) Representative macroscopic images of small intestines. (C) Quantification of small intestinal length. Serum levels of pro‐inflammatory cytokines IL‐6 (D), IL‐1β (E), and TNF‐α (F). Data represent three independent experiments. Quantification of villus height (G), crypt depth (H), and villus height‐to‐crypt depth ratio (I) in distal small intestinal tissue. (J) PCoA plot based on Bray–Curtis dissimilarity (*n* = 4 per group). Small intestine, F value 1.51, *p*‐value 0.107; Cecum, F value 1.93, *p*‐value 0.001; Colon, *F*‐value 1.56, *p*‐value 0.005; number of permutations, 999. (K) Circos diagram illustrating the relative abundance of the top 10 genera across treatment groups. Error bars represent standard error of mean. *p < 0.05; **p < 0.01; ***p < 0.001; ****p < 0.0001; ns, no significance; one‐way ANOVA with Benjamini–Hochberg correction for multiple comparisons.

To assess the therapeutic potential of WMT, we induced intestinal mucositis in mice using 5‐fluorouracil (5‐FU) (Figure 1A) [12]. While FMT displayed limited effect on reversing intestinal length shortening, WMT significantly restored intestinal length (Figure 1B,C). Compared with untreated mice, both FMT and WMT reduced serum IL‐6; however, only WMT significantly lowered IL‐1β and TNF‐α (Figure 1D–F), suggesting a stronger anti‐inflammatory effect than FMT. Histopathological analysis revealed that both interventions efficiently alleviated inflammatory responses, vacuolization, edema, and myeloperoxidase (MPO) positive cells in the distal small intestine (Figure S4). The WMT group exhibited the least disruption of villus length and crypt depth (Figure 1G–I). Together, these data support the enhanced therapeutic efficacy of WMT against intestinal mucositis compared to FMT.

To further investigate the regulatory effects of WMT on gut microbiota, we performed 16S rRNA sequencing analysis of intestinal contents. The results showed that WMT altered gut microbiota diversity, especially in the cecum and colon (Figure 1J, Figure S5), as well as the relative abundance at both the phylum and genus levels (Figure S6A, Figure 1K). The relative abundance of Firmicutes and Bacteroidota changed significantly in the small intestine, cecum, and colon after WMT administration, tending towards levels in the normal group (Figure S6B–D). At the genus level, WMT increased the abundance of beneficial *Lactobacillus* and the butyrate‐producing genus *Intestinimonas* [13], while significantly decreasing pro‐inflammatory taxa, such as *Desulfovibrio* [14], *Lachnoclostridium* [15], and *Tuzzerella* [16] (Figure S6B–D). LEfSe analysis identified differentially abundant taxa between the FMT and WMT groups (Figure S7). In the WMT group, the relative abundance of beneficial Gram‐positive bacteria increased, while that of Gram‐negative bacteria, biofilm‐forming bacteria, and potential pathogens decreased (Figure S8). Overall, these data indicate that WMT reinstates gut microbiota homeostasis and alleviates intestinal mucositis.

## DESIGN OF MICROBIOTA NANOCAPSULES AND THEIR GASTROINTESTINAL ROBUSTNESS

**Figure 2:**
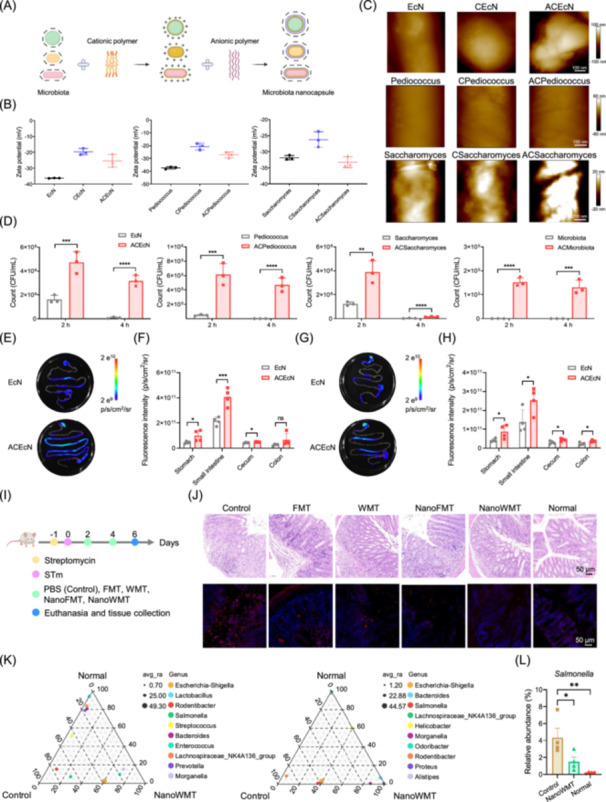
NanoWMT alleviates *Salmonella typhimurium* (STm)‐induced colitis. (A) Schematic of nanocapsule synthesis. (B) Zeta potential of coated EcN, *Pediococcus*, and *Saccharomyces* (*n* = 3). CEcN, EcN coated with chitosan; ACEcN, EcN coated with chitosan‐alginate; CPediococcus, *Pediococcus* coated with chitosan; ACPediococcus, *Pediococcus* coated with chitosan‐alginate; CSaccharomyces, *Saccharomyces* coated with chitosan; ACSaccharomyces, *Saccharomyces* coated with chitosan‐alginate. (C) Representative AFM images of uncoated and coated microbes. Scale bar, 100 nm (EcN, CEcN, ACEcN, *Pediococcus*, CPediococcus, and ACPediococcus) or 200 nm (*Saccharomyces*, CSaccharomyces, and ACSaccharomyces). (D) Quantification analysis of coated EcN, *Pediococcus*, *Saccharomyces*, and microbiota survival after incubation in SGF (*n* = 3 independent experiments, each performed in triplicate). ACMicrobiota, Microbiota coated with chitosan‐alginate. (E, G) Representative IVIS images of EcN in the mouse GI tract after 2 and 4 h post‐gavage. Scale bar, 2 e^9^–2 e^10^ p/s/cm^2^/sr. (F, H) Quantification of fluorescence intensity (*n* = 4). (I) Experimental design and treatment regimen for the STm‐induced colitis model (*n* = 4 or 5). (J) Representative hematoxylin and eosin (H&E) staining (scale bar, 50 μm) and Immunofluorescence images of mCherry‐labeled STm in colon tissue (scale bar, 50 μm). (K) Ternary plot shows genus‐level species distribution in the small intestine and colon. avg_ra, average abundance ratio. (L) Relative abundance of *Salmonella* in the colon. Error bars represent standard error of mean. *p < 0.05; **p < 0.01; ***p < 0.001; ****p < 0.0001; ns, no significance; (D), (F), and (H) were assessed using unpaired *t*‐test (and nonparametric tests); (L) was assessed using one‐way ANOVA (and nonparametric or mixed) with Benjamini–Hochberg correction.

To enhance the therapeutic efficacy of WMT, we engineered nanocapsules for microbiota delivery. Natural bioactive materials are biosafe, biodegradable, and widely explored in tissue engineering and drug delivery systems [17, 18]. As proof of concept, we assembled microbiota nanocapsules via electrostatic layer‐by‐layer deposition of bioactive chitosan (positively charged) and sodium alginate (negatively charged) (Figure 2A). We first evaluated the feasibility of nanocapsules using representative gut microbes, including *Escherichia coli* Nissle 1917 (EcN) (Gram‐negative bacteria, an oral probiotic), *Pediococcus* (Gram‐positive bacteria, used as bio‐preservatives and probiotic candidates), and *Saccharomyces* (fungus, model organism) [17]. Zeta potentials of coated EcN, *Pediococcus,* and *Saccharomyces* are shown in Figure 2B. Due to the positive charge of chitosan and the negative charge of alginate, the zeta potential of coated EcN rose from −36.30 to −19.78 mV, and then dropped to −25.45 mV. Similar trends were observed during the coating process of *Pediococcus* and *Saccharomyces*. Transmission electron microscopy revealed minor morphological changes after coating (Figure S9). Atomic force microscopy images highlighted distinct surface microstructures of the coated microbes compared with the uncoated controls (Figure 2C). Confocal laser scanning microscopy images demonstrated that FITC‐labeled sodium alginate and Rhodamine B‐labeled chitosan successfully coated these representative microbes and gut microbiota (Figures S10, S11). Thus, microbiota nanocapsules were effectively formed via sequential alternating polycation‐polyanion deposition.

We next assessed nanocapsule effects on microbiota gastrointestinal tolerance and colonization. In vitro growth curves of coated EcN, *Pediococcus*, *Saccharomyces*, and whole microbiota closely mirrored those of uncoated controls (Figure S12A–D), suggesting a negligible impact from the chitosan‐alginate multilayer on proliferation. In simulated gastric fluid, coated microbiota survived at significantly higher rates than uncoated forms at 2 and 4 h post‐treatment (Figure 2D), affirming the protective role of nanoshell against gastric stress. In vivo imaging system analysis demonstrated improved survival of mCherry‐labeled coated EcN in the GI tract at 2 h and 4 h post‐oral administration (Figure 2E,G), with the highest fluorescence intensity in the small intestine (Figure 2F,H). Quantitative counts confirmed higher numbers of viable coated EcN across the stomach, small intestine, cecum, and colon compared to uncoated EcN (Figure S12E–H), thereby validating enhanced microbial colonization via nanocapsules.

## EFFICACY OF NANOWMT AGAINST STM INFECTION

We then examined the synergistic therapeutic effect of NanoWMT in an STm‐induced colitis model (Figure 2I) [19]. After treatment with FMT, WMT, and NanoFMT, colon length increased; notably, NanoWMT most effectively alleviated colon shortening (Figure S13A,B). Compared with the control group, all four treatment groups significantly reduced serum inflammatory cytokines (IL‐6, IL‐1β, and TNF‐α), with the NanoWMT group showing the strongest anti‐inflammatory effect (Figure S14). Hematoxylin and eosin staining and MPO immunohistochemistry revealed that all treatments mitigated inflammation, mucosal edema, and MPO levels in the distal colon tissue, with the NanoWMT group demonstrating the greatest improvement (Figure 2J, Figure S15). Immunofluorescence analysis showed that NanoWMT most effectively reduced the fluorescence intensity of mCherry‐labeled STm in the intestinal tract, indicating decreased STm colonization (Figure 2J, Figure S16).

16S rRNA sequencing showed that NanoWMT significantly improved the composition and relative abundance of gut microbiota, and markedly increased the proportions of *Clostridia* (butyrate producer) and *Bacteroides* (Figure 2K, Figures S17–19). These bacteria are beneficial for the treatment of colitis [20]. Importantly, NanoWMT reduced the abundance of harmful bacteria, including Proteobacteria at the phylum level, and *Escherichia‐Shigella* and *Salmonella* at the genus level (Figure 2L, Figures S17–19). The relative abundance of bacterial phenotypes revealed that NanoWMT markedly reduced the proportion of biofilm‐forming bacteria and potential pathogens (Figures S18, S19). Function heatmap analysis suggested that the gut microbiota in the NanoWMT group was enriched in pathways related to replication and repair as well as glycan biosynthesis and metabolism, which may contribute to intestinal mucosa repair (Figure S20). Compared to WMT, NanoWMT was associated with reduced abundance of immune disease‐related pathways (Figure S21), a critical aspect of inflammatory bowel disease pathogenesis. These results underscore the therapeutic potential of NanoWMT in mitigating inflammation and restoring gut microbiota homeostasis, thus providing novel strategies for inflammatory bowel disease management.

Although FMT and SIM transplantation are effective and generally well‐tolerated clinically, both have inherent limitations in comprehensively modulating intestinal microecology [2, 3, 7]. Here, we developed a novel WMT strategy that precisely regulates microbiota homeostasis throughout the entire intestine by simultaneously transplanting SIM and fecal microbiota. WMT demonstrated superior efficacy compared with FMT in reshaping microbial community structure and diversity across the small intestine, cecum, and colon of GF mice. Specifically, WMT enriched beneficial taxa, including *Turicibacter* [8], *Akkermansia* [10], *Faecalibaculum* [10], and *Lachnospiraceae_NK4A136_group* [11], which are essential for host bile acid, short‐chain fatty acid, and lipid metabolism. Encapsulation with biocompatible nanocapsules further potentiated the therapeutic efficacy of WMT by enhancing intestinal colonization and bioavailability of transplanted microbiota. Notably, both WMT and NanoWMT substantially reduced biofilm‐forming bacteria in the small intestine and colon, which may help mitigate bacterial resistance and potentiate antibiotic effectiveness [19]. While our findings establish proof‐of‐concept efficacy in murine models, clinical feasibility and therapeutic efficacy warrant evaluation in future studies.

## AUTHOR CONTRIBUTIONS

**Bufu Tang**: Writing—original draft; visualization; investigation; data curation. **Yuan Cao**: data curation; investigation; visualization; writing—original draft. **Jiasu Li**: Investigation; writing—original draft. **Nan Gao**: Investigation; writing—original draft. **Pingting Gao**: investigation; writing—original draft. **Xiaochao Chen**: Data curation; writing—original draft. **Zunzhen Ming**: Supervision; writing—review and editing. **Zhaoshen Li**: Conceptualization; funding acquisition; writing—review and editing; supervision. **Weiliang Hou**: Conceptualization; funding acquisition; supervision; writing—review and editing. All authors have read the final manuscript and approved it for publication.

## CONFLICT OF INTEREST STATEMENT

The authors declare no conflicts of interest.

## ETHICS STATEMENT

The ethics application was approved by the Research Ethics Committee of Changhai Hospital of Naval Medical University, Shanghai, China (CHEC(A.E)2025‐051).

## Supporting information


**Figure S1.** WMT restores intestinal microbiota in small intestine, cecum, and colon of GF mice.
**Figure S2.** The β diversity and LEfSe analysis between the FMT group and the WMT group in GF mice.
**Figure S3.** Functional prediction analysis of gut microbiota using PICRUSt in the colon contents of GF mice.
**Figure S4.** H&E staining and MPO immunohistochemistry of distal small intestine.
**Figure S5.** Comparison of the alpha‐diversity index (Chao1 and ACE) between the FMT group and the WMT group.
**Figure S6.** Gut microbiota composition in 5‐FU‐induced mucositis mice.
**Figure S7.** LEfSe analysis identified bacterial taxa with differential abundance between the FMT group and the WMT group in 5‐FU‐induced mucositis mice.
**Figure S8.** The heatmap of phenotypic abundance.
**Figure S9.** TEM images of microbes.
**Figure S10.** CLSM images of coated EcN, *Pediococcus*, and *Saccharomyces*.
**Figure S11.** CLSM images of the coated whole microbiota.
**Figure S12.** Gastrointestinal resistance of encapsulated microbiota.
**Figure S13.** NanoWMT treatment significantly alleviated the shortening of colon length.
**Figure S14.** Serum levels of IL‐6, IL‐1β, and TNF‐α.
**Figure S15.** MPO immunohistochemistry of distal colon tissue.
**Figure S16.** Immunofluorescence images of mCherry‐labeled STm in the small intestine and cecum.
**Figure S17.** NanoWMT reduces the harmful bacteria abundance in the small intestinal contents of STm‐induced colitis mice.
**Figure S18.** Bacterial composition and differential abundance of the cecal contents in STm‐induced colitis mice.
**Figure S19.** Bacterial composition and differential abundance of the colonic contents in STm‐induced colitis mice.
**Figure S20.** Heatmap of functional abundance.
**Figure S21.** Functional prediction analysis of gut microbiota based on PICRUSt in the colon of STm‐induced colitis mice.

## Data Availability

The data that support the findings of this study are openly available in GitHub at https://github.com/WYQHWL/NanoWMT. All data supporting the findings of this study are available within the article and its supplementary information files. Supplementary materials (methods, figures, graphical abstract, slides, videos, Chinese translated version, and update materials) may be found in the online DOI or iMeta Science http://www.imeta.science/.

## References

[1] Gilbert, Jack A. , Martin J. Blaser , J. Gregory Caporaso , Janet K. Jansson , Susan V. Lynch , and Rob Knight . 2018. “Current Understanding of the Human Microbiome.” Nature Medicine 24: 392–400. 10.1038/nm.4517 PMC7043356 29634682

[2] Allegretti, Jessica R. , Sahil Khanna , Benjamin H. Mullish , and Paul Feuerstadt . 2024. “The Progression of Microbiome Therapeutics for the Management of Gastrointestinal Diseases and Beyond.” Gastroenterology 167: 885–902. 10.1053/j.gastro.2024.05.004 38754739

[3] Wang, Xinjun , Di Zhao , Dexi Bi , Long Li , Hongliang Tian , Fang Yin , Tao Zuo , et al. 2025. “Fecal Microbiota Transplantation: Transitioning From Chaos and Controversial Realm to Scientific Precision Era.” Science Bulletin 70: 970–985. 10.1016/j.scib.2025.01.029 39855927

[4] DeLeon, Orlando , Mora Mocanu , Alan Tan , Ashley M. Sidebottom , Jason Koval , Hugo D. Ceccato , Sarah Kralicek , et al. 2025. “Microbiome Mismatches from Microbiota Transplants Lead to Persistent Off‐Target Metabolic and Immunomodulatory Effects.” Cell 188: 3927–3941.e13. 10.1016/j.cell.2025.05.014 40482640 PMC12330209

[5] Kastl, Arthur J. , Natalie A. Terry , Gary D. Wu , and Lindsey G. Albenberg . 2020. “The Structure and Function of the Human Small Intestinal Microbiota: Current Understanding and Future Directions.” Cellular and Molecular Gastroenterology and Hepatology 9: 33–45. 10.1016/j.jcmgh.2019.07.006 31344510 PMC6881639

[6] Yersin, Simon , and Pascale Vonaesch . 2024. “Small Intestinal Microbiota: from Taxonomic Composition to Metabolism.” Trends in Microbiology 32: 970–983. 10.1016/j.tim.2024.02.013 38503579

[7] Ye, Chen , Zhiliang Lin , Jiaqu Cui , Xiaoqiong Lv , Shaoyi Zhang , Chunlian Ma , Yinmei Yan , et al. 2022. “Fecal Microbiota and Human Intestinal Fluid Transplantation: Methodologies and Outlook.” Frontiers in Medicine 9: e830004. 10.3389/fmed.2022.830004 PMC9158325 35665355

[8] Lynch, Jonathan B. , Erika L. Gonzalez , Kayli Choy , Kym F. Faull , Talia Jewell , Abelardo Arellano , Jennifer Liang , et al. 2023. “Gut Microbiota Turicibacter Strains Differentially Modify Bile Acids and Host Lipids.” Nature Communications 14: 3669. 10.1038/s41467-023-39403-7 PMC10281990 37339963

[9] Li, Chaoliang , Panrui Zhang , Yadong Xie , Shishan Wang , Meng Guo , Xiaowei Wei , Kaiguang Zhang , et al. 2024. “Enterococcus‐Derived Tyramine Hijacks α2A‐Adrenergic Receptor in Intestinal Stem Cells to Exacerbate Colitis.” Cell Host & Microbe 32: 950–963. 10.1016/j.chom.2024.04.020 38788722

[10] Martín, Rebeca , David Rios‐Covian , Eugénie Huillet , Sandrine Auger , Sarah Khazaal , Luis G. Bermúdez‐Humarán , Harry Sokol , Jean‐Marc Chatel , and Philippe Langella . 2023. “Faecalibacterium: A Bacterial Genus With Promising Human Health Applications.” FEMS Microbiology Reviews 47: 1–18. 10.1093/femsre/fuad039 PMC10410495 37451743

[11] Huang, Shuangbo , Jianzhao Chen , Zhijuan Cui , Kaidi Ma , Deyuan Wu , Jinxi Luo , Fuyong Li , et al. 2023. “Lachnospiraceae‐Derived Butyrate Mediates Protection of High Fermentable Fiber Against Placental Inflammation in Gestational Diabetes Mellitus.” Sci Adv 9: eadi7337. 10.1126/sciadv.adi7337 37922350 PMC10624355

[12] Kim, Kyung‐Ah , Makoto Kakitani , Jingsong Zhao , Takeshi Oshima , Tom Tang , Minke Binnerts , Yi Liu , et al. 2005. “Mitogenic Influence of Human R‐Spondin1 on the Intestinal Epithelium.” Science 309: 1256–1259. 10.1126/science.1112521 16109882

[13] Rampanelli, Elena , Nadia Romp , Antonio Dario Troise , Jakshana Ananthasabesan , Hao Wu , Ismail Sahin Gül , Sabrina De Pascale , et al. 2025. “Gut Bacterium Intestinimonas Butyriciproducens Improves Host Metabolic Health: Evidence From Cohort and Animal Intervention Studies.” Microbiome 13: 15. 10.1186/s40168-024-02002-9 39833973 PMC11744835

[14] Xie, Runxiang , Yu Gu , Mengfan Li , Lingfeng Li , Yunwei Yang , Yue Sun , Bingqian Zhou , et al. 2024. “Desulfovibrio Vulgaris Interacts With Novel Gut Epithelial Immune Receptor LRRC19 and Exacerbates Colitis.” Microbiome 12: 4. 10.1186/s40168-023-01722-8 38172943 PMC10763354

[15] Gao, Han , Mingming Sun , Ai Li , Qiaoyan Gu , Dengfeng Kang , Zhongsheng Feng , Xiaoyu Li , et al. 2025. “Microbiota‐Derived IPA Alleviates Intestinal Mucosal Inflammation Through Upregulating Th1/Th17 Cell Apoptosis in Inflammatory Bowel Disease.” Gut Microbes 17: e2467235. 10.1080/19490976.2025.2467235 PMC11834480 39956891

[16] Yu, Zuoting , Dinggang Li , and Hongxiang Sun . 2023. “Herba Origani Alleviated DSS‐induced Ulcerative Colitis in Mice through Remolding Gut Microbiota to Regulate Bile Acid and Short‐Chain Fatty Acid Metabolisms.” Biomedicine & Pharmacotherapy 161: e114409. 10.1016/j.biopha.2023.114409 36822021

[17] Zhou, Jun , Maoyi Li , Qiufang Chen , Xinjie Li , Linfu Chen , Ziliang Dong , Wenjun Zhu , et al. 2022. “Programmable Probiotics Modulate Inflammation and Gut Microbiota for Inflammatory Bowel Disease Treatment After Effective Oral Delivery.” Nature Communications 13: e3432. 10.1038/s41467-022-31171-0 PMC9198027 35701435

[18] Hou, Weiliang , Weifeng Hong , Songhua Cai , Dandan Guo , Zhiping Yan , Jinyu Zhu , Yang Shen , et al. 2025. “RRM2‐Targeted Nanocarrier Enhances Radiofrequency Ablation Efficacy in Hepatocellular Carcinoma Through Ferroptosis Amplification and Immune Remodeling.” iMeta 4: e70067. 10.1002/imt2.70067 41112038 PMC12527986

[19] Hou, Weiliang , Yuan Cao , Jifeng Wang , Fang Yin , Jiahui Wang , Ning Guo , Ziyi Wang , et al. 2025. “Single‐Cell Nanocapsules of Gut Microbiota Facilitate Fecal Microbiota Transplantation.” Theranostics 15: 2069–2084. 10.7150/thno.104852 39897545 PMC11780513

[20] Fan, Jingjing , Ying Wu , Xing Wang , Habib Ullah , Zhenmin Ling , Pu Liu , and Yu Wang , et al. 2025. “The Probiotic Enhances Donor Microbiota Stability and Improves the Efficacy of Fecal Microbiota Transplantation for Treating Colitis.” Journal of Advanced Research. In press. 10.1016/j.jare.2025.03.017 40089059

