# Supplementary material for: Whole microbiota transplantation restores gut homeostasis throughout the gastrointestinal tract
Source: Imeta. 2025 Nov 11;4(6):e70091. doi: 10.1002/imt2.70091 (PMC12747539; doi:10.1002/imt2.70091)
Supplement: Supplementary file 1 — Figure S1. WMT restores intestinal microbiota in small intestine, cecum, and colon of GF mice. Figure S2. The β diversity and LEfSe analysis between the FMT group and the WMT group in GF mice. Figure S3. Functional prediction analysis of gut microbiota using PICRUSt in the colon contents of GF mice. Figure S4. H&E staining and MPO immunohistochemistry of distal small intestine. Figure S5. Comparison of the alpha‐diversity index (Chao1 and ACE) between the FMT group and the WMT group. Figure S6. Gut microbiota composition in 5‐FU‐induced mucositis mice. Figure S7. LEfSe analysis identified bacterial taxa with differential abundance between the FMT group and the WMT group in 5‐FU‐induced mucositis mice. Figure S8. The heatmap of phenotypic abundance. Figure S9. TEM images of microbes. Figure S10. CLSM images of coated EcN, Pediococcus, and Saccharomyces. Figure S11. CLSM images of the coated whole microbiota. Figure S12. Gastrointestinal resistance of encapsulated microbiota. Figure S13. NanoWMT treatment significantly alleviated the shortening of colon length. Figure S14. Serum levels of IL‐6, IL‐1β, and TNF‐α. Figure S15. MPO immunohistochemistry of distal colon tissue. Figure S16. Immunofluorescence images of mCherry‐labeled STm in the small intestine and cecum. Figure S17. NanoWMT reduces the harmful bacteria abundance in the small intestinal contents of STm‐induced colitis mice. Figure S18. Bacterial composition and differential abundance of the cecal contents in STm‐induced colitis mice. Figure S19. Bacterial composition and differential abundance of the colonic contents in STm‐induced colitis mice. Figure S20. Heatmap of functional abundance. Figure S21. Functional prediction analysis of gut microbiota based on PICRUSt in the colon of STm‐induced colitis mice. [file IMT2-4-e70091-s001.docx]

**Supporting information to**

**Whole Microbiota Transplantation Restores Gut Homeostasis Throughout the Gastrointestinal Tract**

**Running title:** WMT for restoring gut microbiota homeostasis

Bufu Tang^1#^, Yuan Cao^2#^, Jiasu Li^3#^, Nan Gao^3#^, Pingting Gao^4^, Xiaochao Chen^5^, Zunzhen Ming^6*^, Zhaoshen Li^3*^, Weiliang Hou^3*^

^1^Department of Interventional Radiology, Zhongshan hospital, Shanghai Institute of Medical Imaging, Shanghai, National Clinical Research Center of Interventional Medicine, Fudan University, Shanghai, 200032, China

^2^Institute of Clinical Science, Zhongshan Hospital, Fudan University, Shanghai, 200032, China

^3^Department of Gastroenterology, Shanghai Institute of Pancreatic Diseases, National Key Laboratory of Immunity and Inflammation, Changhai Clinical Research Unit, Changhai Hospital, Naval Medical University, Shanghai, 200433, China

^4^Shanghai Collaborative Innovation Center of Endoscopy, Endoscopy Center and Endoscopy Research Institute of Zhongshan Hospital, Fudan University, Shanghai, 200032, China

^5^Department of Proctology, Chengdu Anorectal Hospital, Chengdu, 500643, China

^6^School of Life Sciences, Shanghai University, Shanghai, 200444, China

^#^These authors contributed equally: Bufu Tang, Yuan Cao, Jiasu Li, Nan Gao

^*^Correspondence: houweiliang@tongji.edu.cn (Weiliang Hou); zhsl@vip.163.com (Zhaoshen Li); mingzunzhen@shu.edu.cn (Zunzhen Ming)

**METHODS**

**Microbes and medium**

The representative Gram-negative bacteria used in this study was *Escherichia coli* Nissle 1917 (EcN), purchased from the China General Microbiological Culture Collection Center (CGMCC, China) and cultured at 37 °C in Luria-Bertani (LB) medium. *Pediococcus acidilactici* DQ2 was a typical Gram-positive bacterium, stored at CGMCC with registration number 7471 and grown in Man-Rogosa-Sharpe (MRS) medium. The representative fungus used in this study was *Saccharomyces*, cultured in Yeast Extract Peptone Dextrose (YPD) medium.

**Microbiota preparation**

Fresh mouse feces were collected in sterile tube, added sterile PBS, and then homogenized manually using a pipette. The suspension was filtered through a 70 μm nylon filter to remove large particles and fibrous substances to obtain fecal microbiota for FMT.

Small intestinal fluid was collected from all small intestinal segment, followed by filtration (70 μm nylon filter), centrifugation (8000 g × 5 min) and resuspension with an equal amount of PBS to obtain small intestinal microbiota. The microbiota for WMT was prepared by fecal microbiota and small intestinal microbiota based on their bacterial colony density in real GI tract [[1](#_ENREF_1)]. Freshly prepared microbiota was stored at 4 ℃ and used for subsequent experiments within 3 days.

**Nanocapsules synthesis**

1 mL of EcN (2 × 10^8^ CFU/mL), *Pediococcus* (5 × 10^7^ CFU/mL), *Saccharomyces* (2 × 10^7^ CFU/mL), and microbiota (2 × 10^6^ CFU/mL) were centrifugated and resuspended in PBS. Then, 0.125 mL of chitosan (1 mg/mL dissolved in 0.2% acetic acid with pH 6.0) was added and gently shook for 10 min. These microbes were washed and resuspended in PBS, followed by adding 0.125 mL of sodium alginate (2 mg/mL), and performed slight shaking (10 min) to obtain the final nanocapsules. The recovery rate of EcN, *Pediococcus, Saccharomyces,* and microbiota was 87.3%, 84.0%, 82.0%, and 77.7% after nanocoating, respectively.

**Nanocapsules characterization**

Microbes labeled with DAPI, chitosan labeled with Rhodamine B, and sodium alginate labeled with FITC were observed by a laser scanning confocal microscope (Leica TCS SP8, Germany). The morphology of microbes was visualized using a transmission electron microscope (Hitachi, Japan) and an atomic force microscope (Dimension FastScan Bio, Bruker, USA).

**In vitro survival and growth determination**

Equal amounts of bare microbes or nanocapsules coated microbes were resuspended in 1 mL of medium containing simulated SGF with 10 g/L pepsin in 0.85% NaCl solution (HCl, pH 2.5) and counted on plates at predetermined time points. EcN, *Pediococcus*, *Saccharomyces*, microbiota and their derivatives were respectively cultured in LB medium, YPD medium, MRS medium and GAM medium at 37 ℃ for plate count to measure the growth curve.

**Animals**

Balb/c mice (male, 6-8 weeks old) were purchased from Beijing Vital River Laboratory Animal Technology Co., Ltd. GF KM mice (male, 6-8 weeks old) were obtained from the Department of Laboratory Animal Science of the Tenth People’s Hospital of Tongji University and bred in a gnotobiotic environment. All the animal experiment procedures were carried out in accordance with the Shanghai Medical Experimental Animal Care guidelines. All animal protocols were reviewed and approved by the Ethics Committee of Changhai Hospital of Naval Medical University, Shanghai, China.

**Microbiota colonization of GF mice**

The GF KM mice were orally administered with 2 × 10^6^ CFU of FMT or WMT. After 24 h, mice were sacrificed and intestinal samples were collected for 16S rRNA sequencing.

**Oral bioavailability in SPF mice**

Balb/c mice were gavaged with 2 × 10^8^ CFU EcN or ACEcN. At 2 h or 4 h post-administration, the mice were sacrificed and their intestines were harvested for imaging by the in vivo imaging system (IVIS Lumina II, Caliper).

**5-FU-induced mucositis model**

The 5-FU-induced mouse intestinal mucositis model was established based on our previous study [[2](#_ENREF_2)]. Briefly, Balb/c mice were intraperitoneally injected with 5-FU (150 mg/kg per mouse), and then from the day of random grouping, these mice were orally administered with PBS, FMT or WMT (2 × 10^8^ CFU/mouse/day) for 5 days. Mice treated with PBS were served as the control group. Serum samples and intestinal tissues were obtained for measurement of inflammatory cytokines and histopathology analysis, respectively.

**STm-induced colitis model**

The STm infection was established as described previously [[3-5](#_ENREF_3)]. Before infection, Balb/c mice were treated with 100 μL of streptomycin solution (200 mg/mL), and then orally inoculated with 5 × 10^7^ CFU of *Salmonella*. At 2 days and 4 days post-infection, the mice were treated with the same dose of microbiota or Nanomicrobiota. Mice were weighted every day and sacrificed at 6 days post-infection. Samples of the inflamed colon from the mice were taken for blind histopathological analysis and blood samples were collected for cytokine levels detection using ELISA kits.

**Histopathology analysis**

Tissue samples were fixed in 4% formalin, embedded in paraffin according to standard procedures, and then cut into 4 μm sections for H&E staining and MPO immunohistochemical staining. All the images of the tissues were captured by the 3D HISTECH Pannoramic 250 (3DHISTECH, Hungary). The activities of MPO were evaluated through MPO staining.

**Gut microbiota profiling**

The sequencing and analysis of the 16S rRNA gene were carried out by the GENE DENOVO Technologies (Guangzhou, China). The total genomic DNA was extracted from the intestinal contents, and sequenced by constructing sequencing libraries on Illumina HiSeq 2500. The original image data files obtained by high-throughput sequencing were converted into Sequenced Reads after Base Calling analysis. Taxonomic analysis was performed on samples at various taxonomic levels to obtain the community structure at the phylum and genus levels. Bioinformatics analysis of the gut microbiota was conducted using the cloud platform (https://www.omicsmart.com).

**Statistical analysis**

The statistical analyses were performed using IBM SPSS Statistics (version 26.0) or GraphPad Prism (version 9.5) and noted in figure legends. Data description employed mean and standard error. The two independent groups were compared using the unpaired *t*-test (and nonparametric tests), while the comparisons of multiple groups were conducted using one-way ANOVA (and nonparametric or mixed), with Benjamini-Hochberg correction applied. The *p*-value less than 0.05 was considered to be statistically significant.


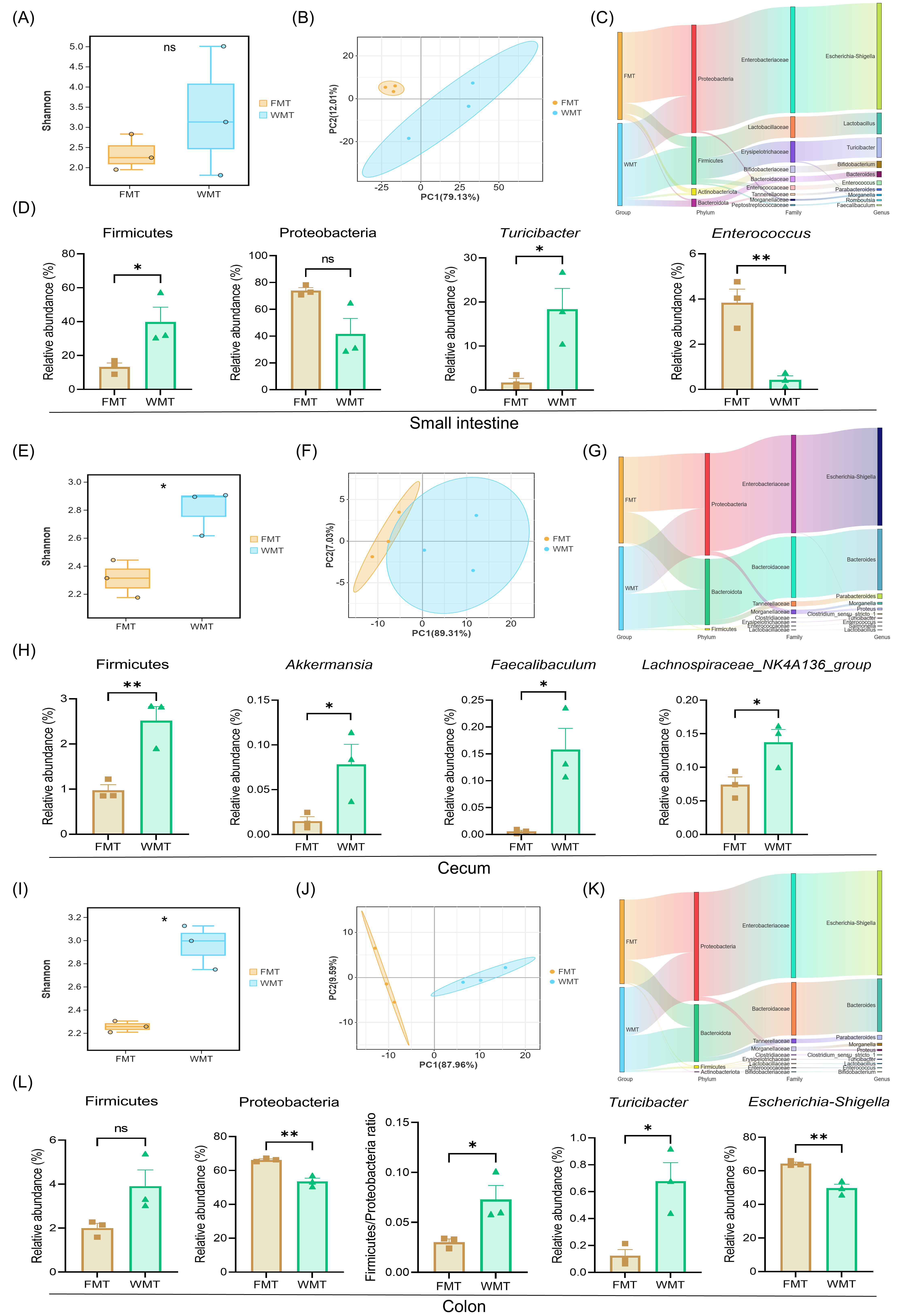


**Figure S1** **WMT restores intestinal microbiota in small intestine, cecum and colon of GF mice.** (A, B) Alpha diversity (A) and beta diversity (B) of gut microbiota in small intestine. F value, 3.63; *p* value, 0.1; number of permutations, 999. (C) Sankey plot shows the dynamic changes in the bacterial taxonomic composition at the phylum, family, and genus levels in the small intestine. (D) Relative abundance of representative microbiota in small intestine. (E, F) Alpha diversity (E) and beta diversity (F) of gut microbiota in cecum. F value, 8.34; *p* value, 0.1; number of permutations, 999. (G) Bacterial taxonomic composition at the phylum, family, and genus levels in cecum. (H) Relative abundance of representative microbiota in cecum. (I, J) Alpha diversity (I) and beta diversity (J) of gut microbiota in colon. F value, 12.62; *p* value, 0.1; number of permutations, 999. (K) Bacterial taxonomic composition at the phylum, family, and genus levels in colon. (L) Relative abundance of representative microbiota in colon. Error bars represent standard error of mean (n = 3). ^*^*p* < 0.05; ^**^*p* < 0.01; ns, no significance; (A), (E) and (I) were assessed using Wilcoxon test; (D), (H) and (L) were assessed using unpaired *t*-test (and nonparametric tests).


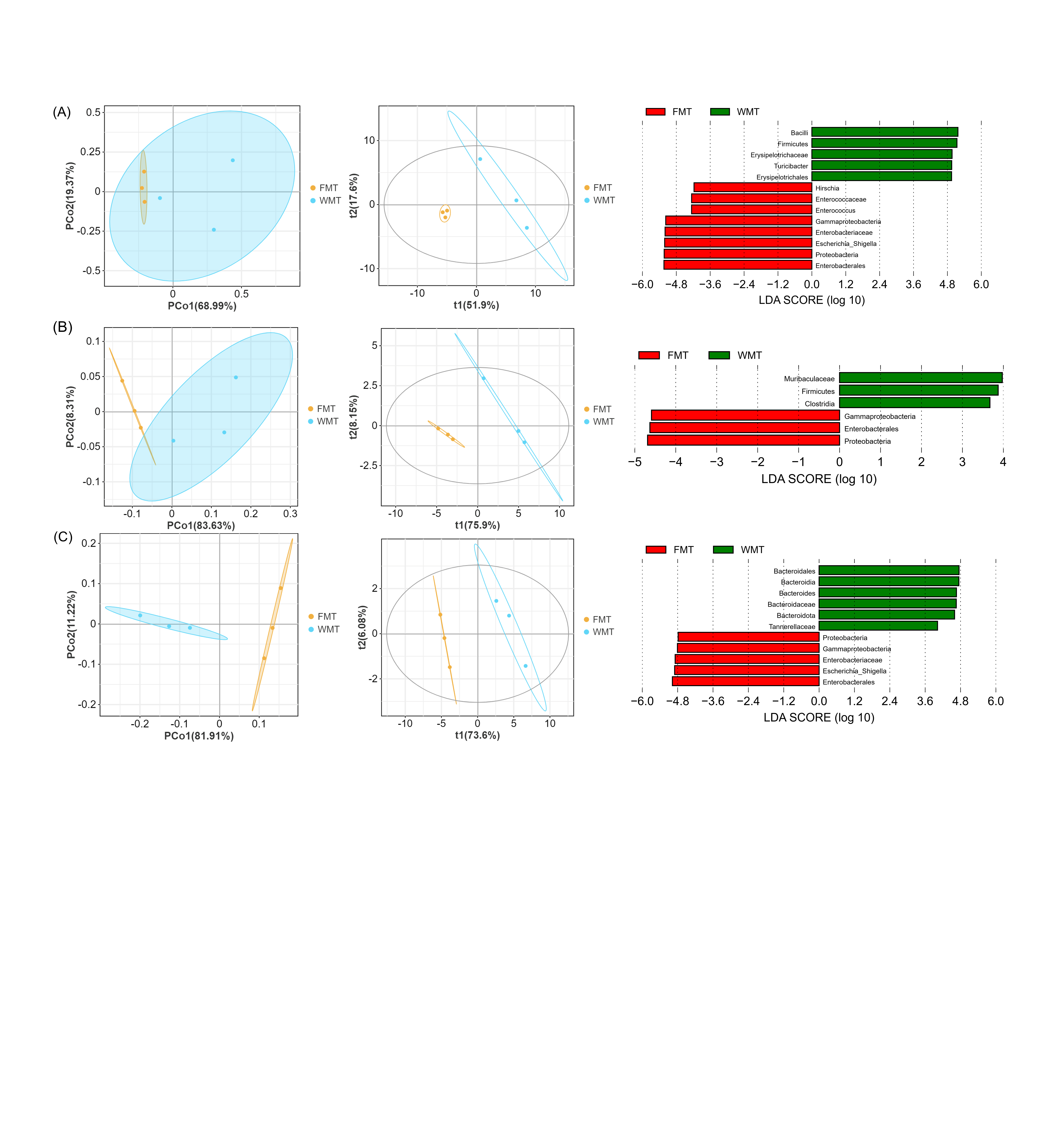


**Figure S2** **The β diversity and LEfSe analysis between the FMT group and the WMT group in GF mice.** PCoA (left) and PLS-DA (middle) plots at the OTU level based on Bray-Curtis distance (n = 3). (A) Small intestine, LDA score > 4. Left and middle, F value 3.63, *p* value 0.1, number of permutations 999. (B) Cecum, LDA > 3.5. Left and middle, F value 8.34, *p* value 0.1, number of permutations 999. (C) Colon, LDA > 4. Left and middle, F value 12.62, *p* value 0.1, number of permutations 999.


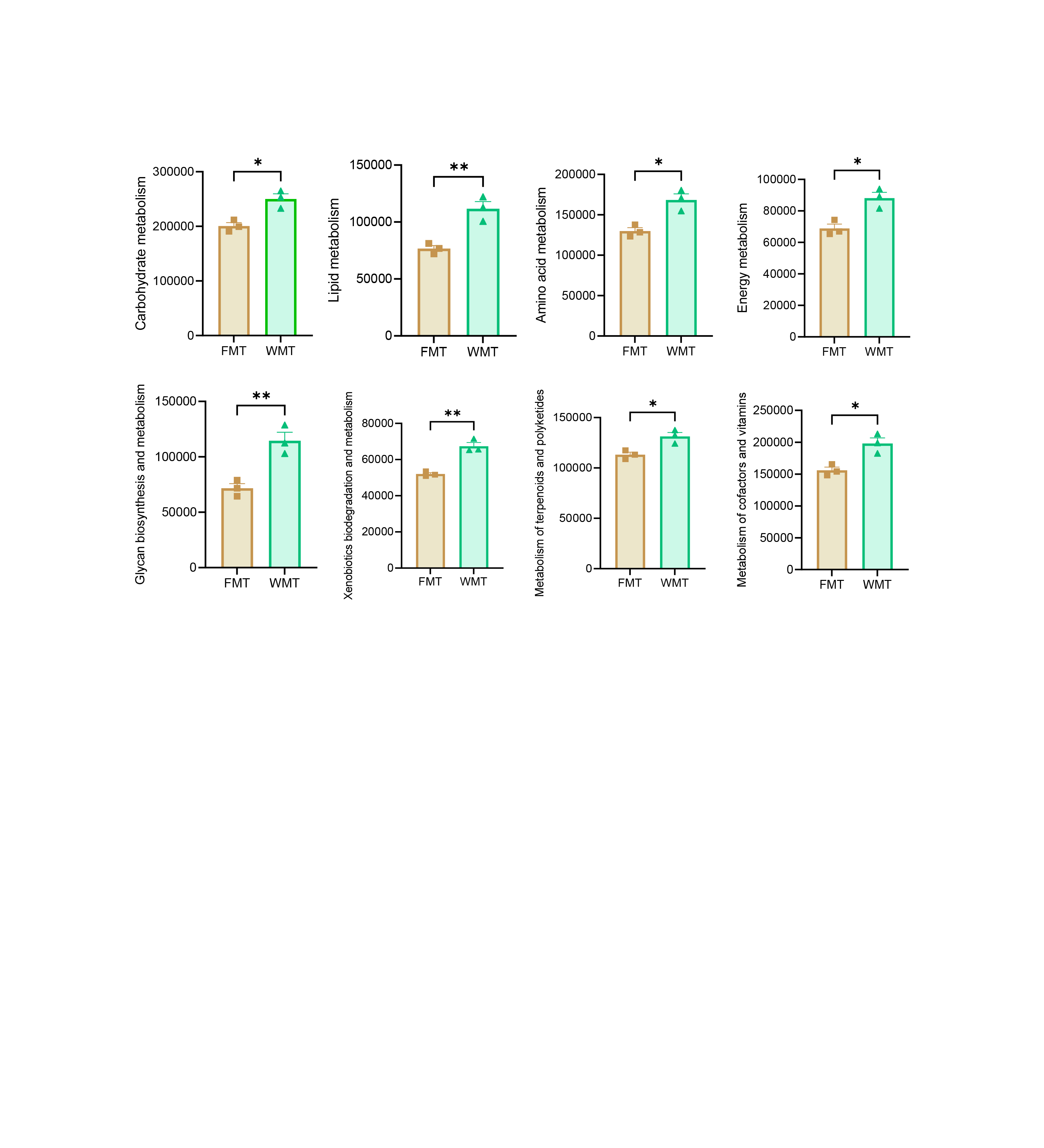


**Figure S3** **Functional prediction analysis of gut microbiota using PICRUSt in the colon contents of GF mice.** Error bars represent standard error of mean (n = 3). ^*^*p* < 0.05; ^**^*p* < 0.01; unpaired *t*-test (and nonparametric tests).


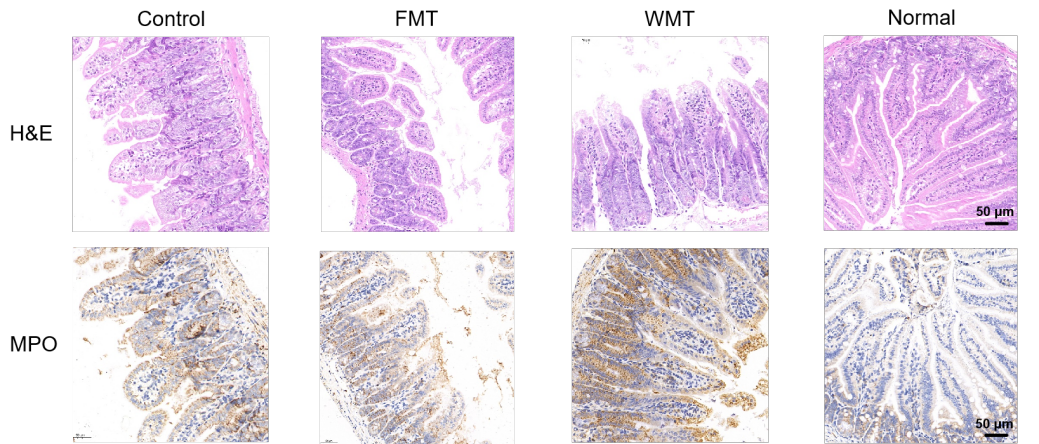


**Figure S4** **H&E staining and MPO immunohistochemistry of distal small intestine.** Tissue samples were obtained on the fifth day of the experiment (scale bar, 50 μm).


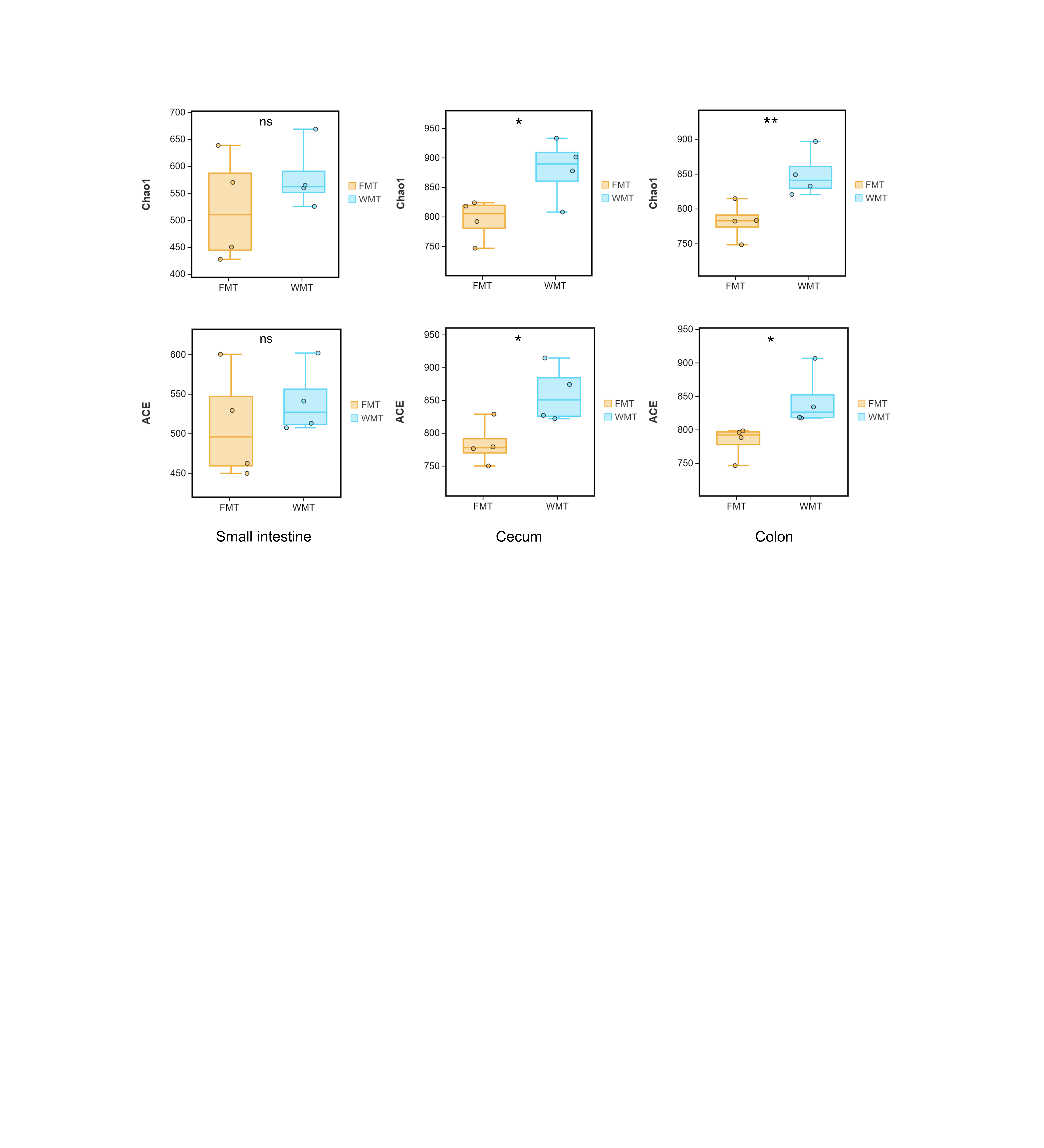


**Figure S5** **Comparison of the alpha-diversity index (Chao1 and ACE) between the FMT group and the WMT group.** Error bars represent standard error of mean (n = 4). ^*^*p* < 0.05; ^**^*p* < 0.01; ns, no significance; Wilcoxon test.


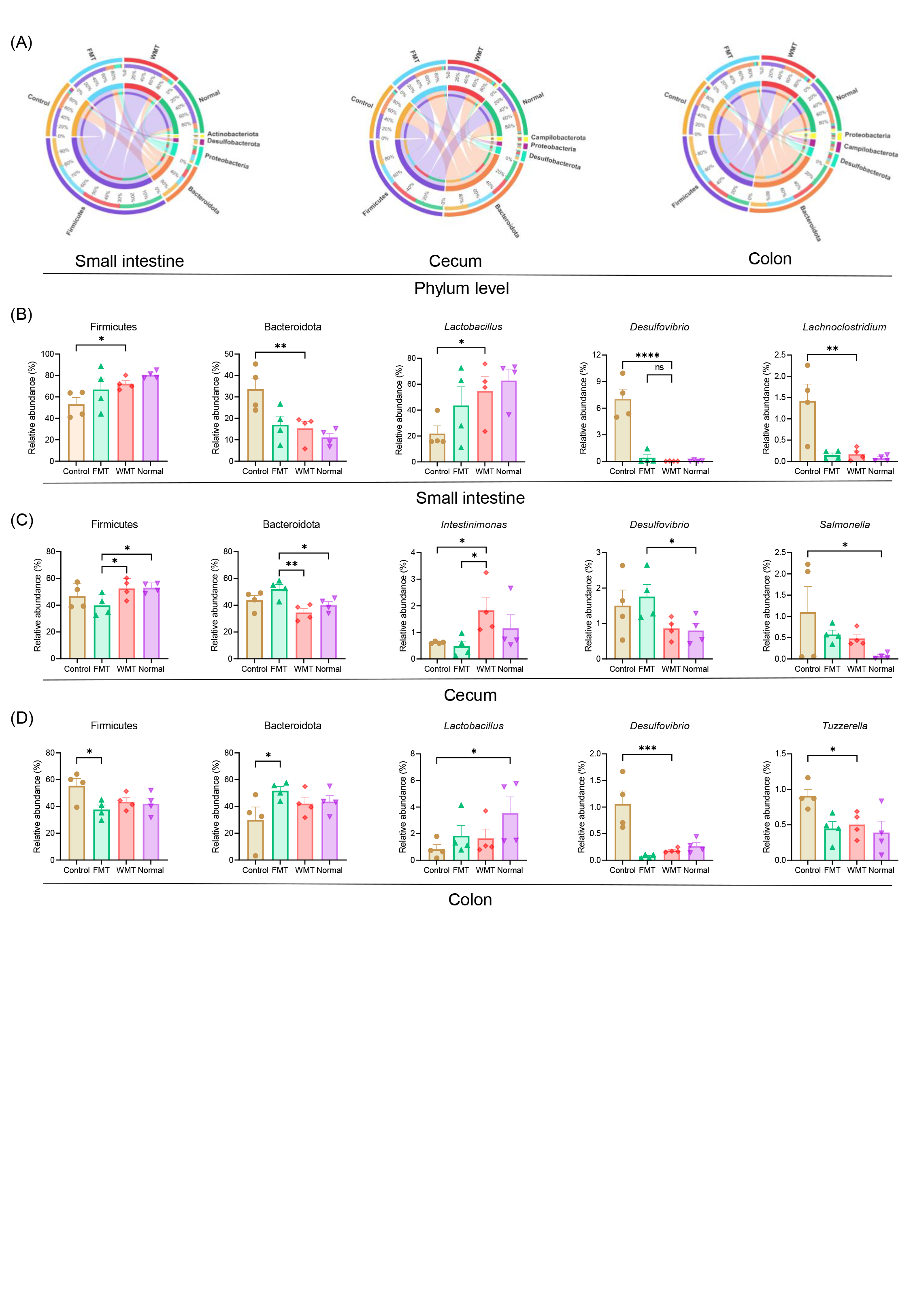


**Figure S6** **Gut microbiota composition in 5-FU-induced mucositis mice.** (A) Circos diagram illustrating relative abundance of top 5 phyla. (B-D) Representatives of microbiota at the phylum and genus levels in small intestine, cecum, and colon. Error bars represent standard error of mean (n = 4). ^*^*p* < 0.05; ^**^*p* < 0.01; ^***^*p* < 0.001; ^****^*p* < 0.0001; ns, no significance; one-way ANOVA (and nonparametric or mixed) with Benjamini-Hochberg correction.


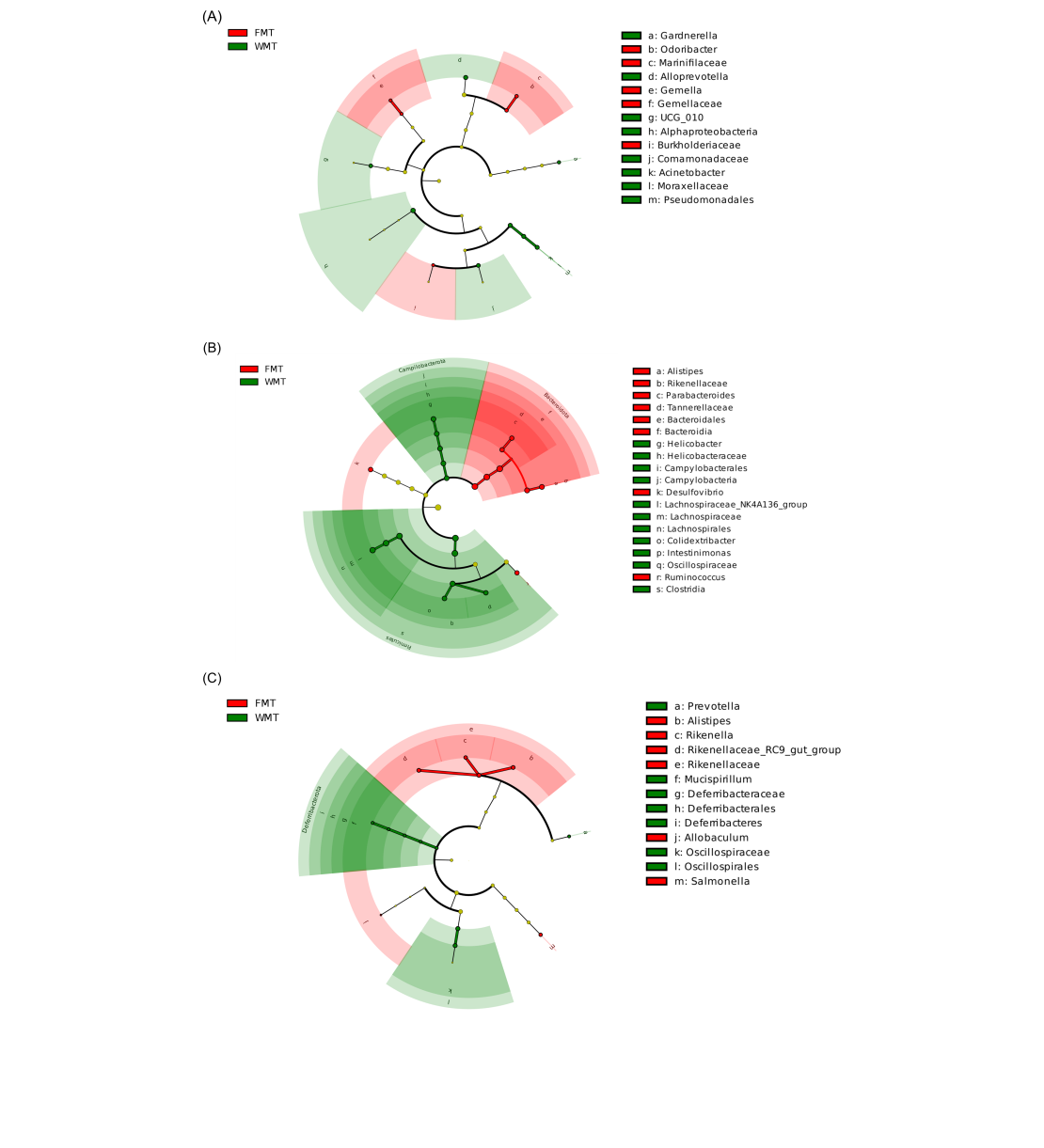


**Figure S7** **LEfSe analysis identified bacterial taxa with differential abundance between the FMT group and the WMT group in 5-FU-induced mucositis mice.** (n = 4) (A) Small intestine, LDA > 2. (B) Cecum, LDA > 3.5. (C) Colon, LDA > 2.5.


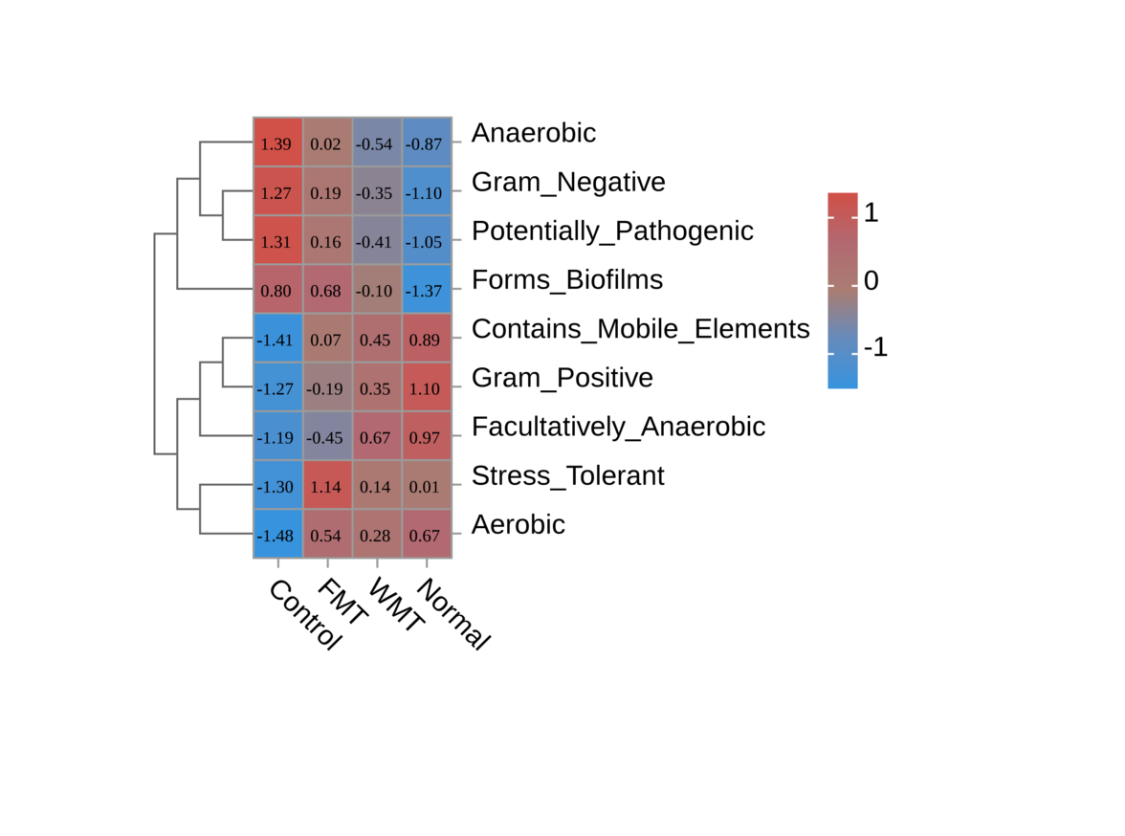


**Figure S8** **The heatmap of phenotypic abundance.** To predict the relative abundance of bacterial phenotypes in the small intestine of mice with 5-FU-induced mucositis.


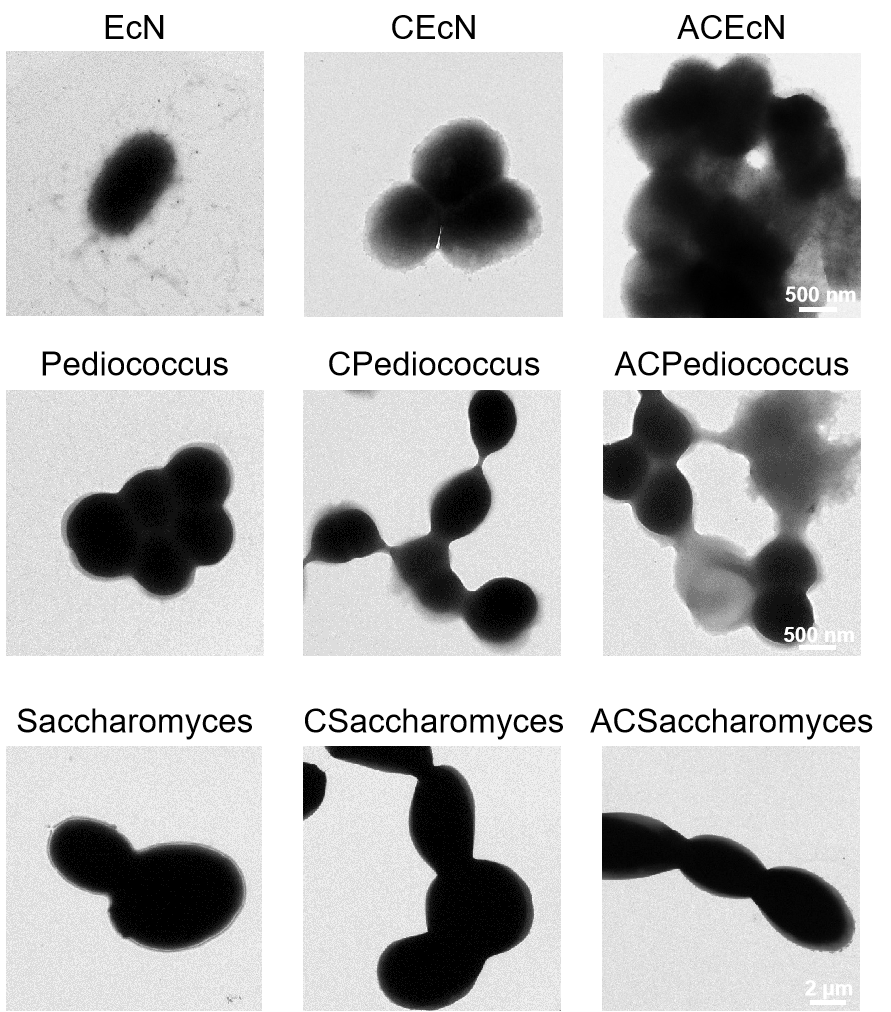


**Figure S9** **TEM images of microbes.** Scale bar, 500 nm (EcN, CEcN, ACEcN, *Pediococcus*, CPediococcus, and ACPediococcus) or 2 μm (*Saccharomyces*, CSaccharomyces and ACSaccharomyces).

**
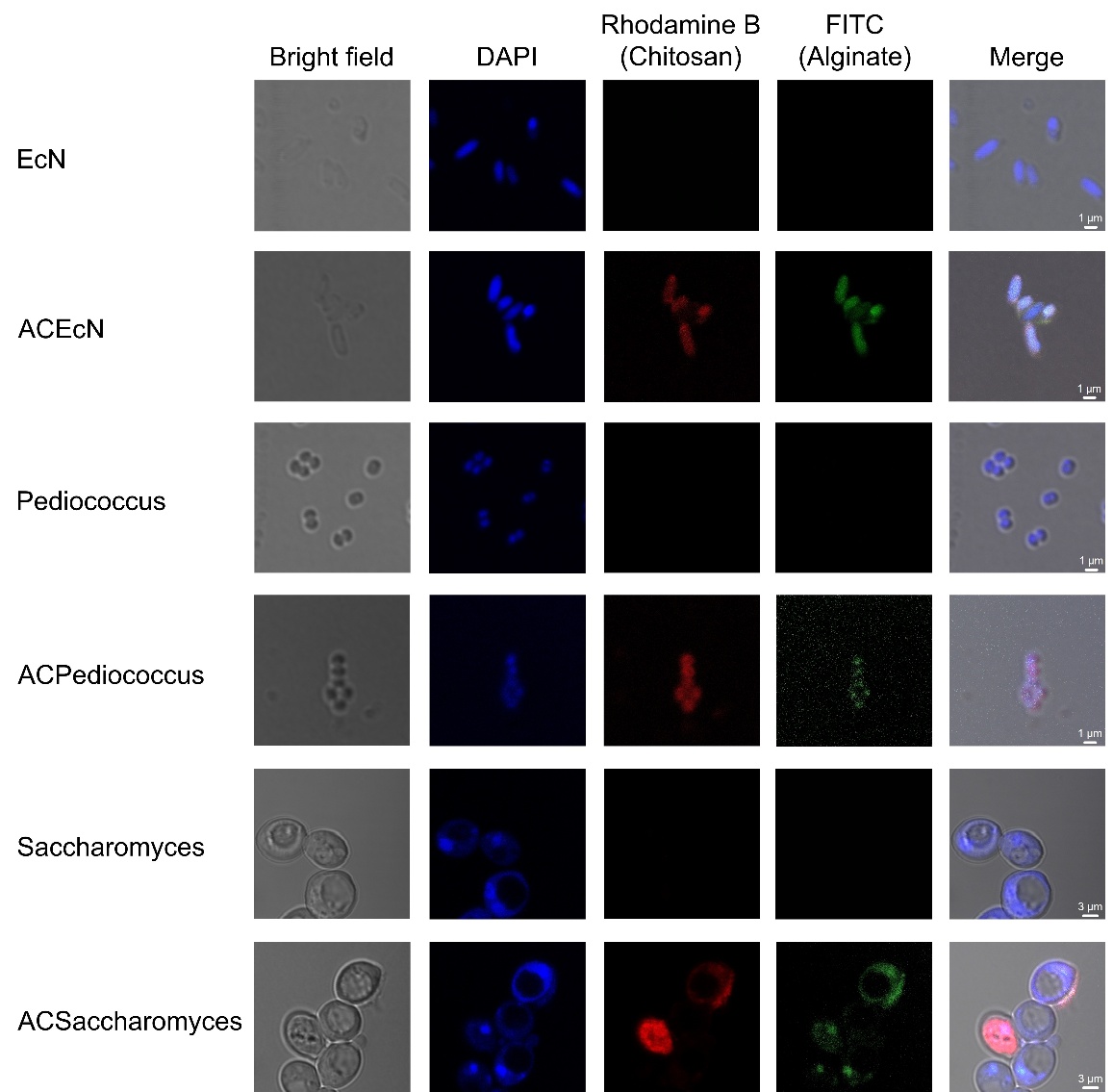
**

**Figure S10** **CLSM images of coated EcN, *Pediococcus*, and *Saccharomyces*.** Microbes were labeled with DAPI, chitosan with Rhodamine B, and sodium alginate with FITC. Scale bar, 1 μm (EcN, ACEcN, *Pediococcus*, and ACPediococcus) or 3 μm (*Saccharomyces* and ACSaccharomyces).


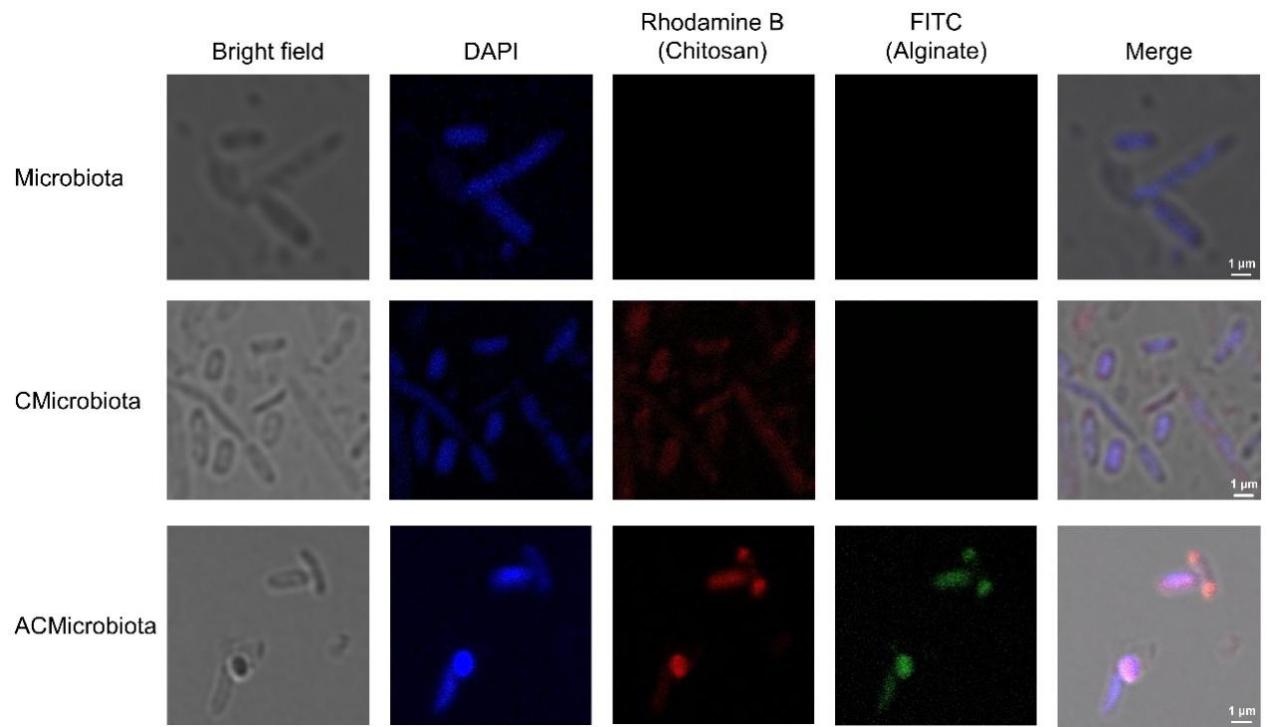


**Figure S11** **CLSM images of the coated whole microbiota.** Microbes were labeled with DAPI, chitosan with Rhodamine B, and sodium alginate with FITC (scale bar, 1 μm).


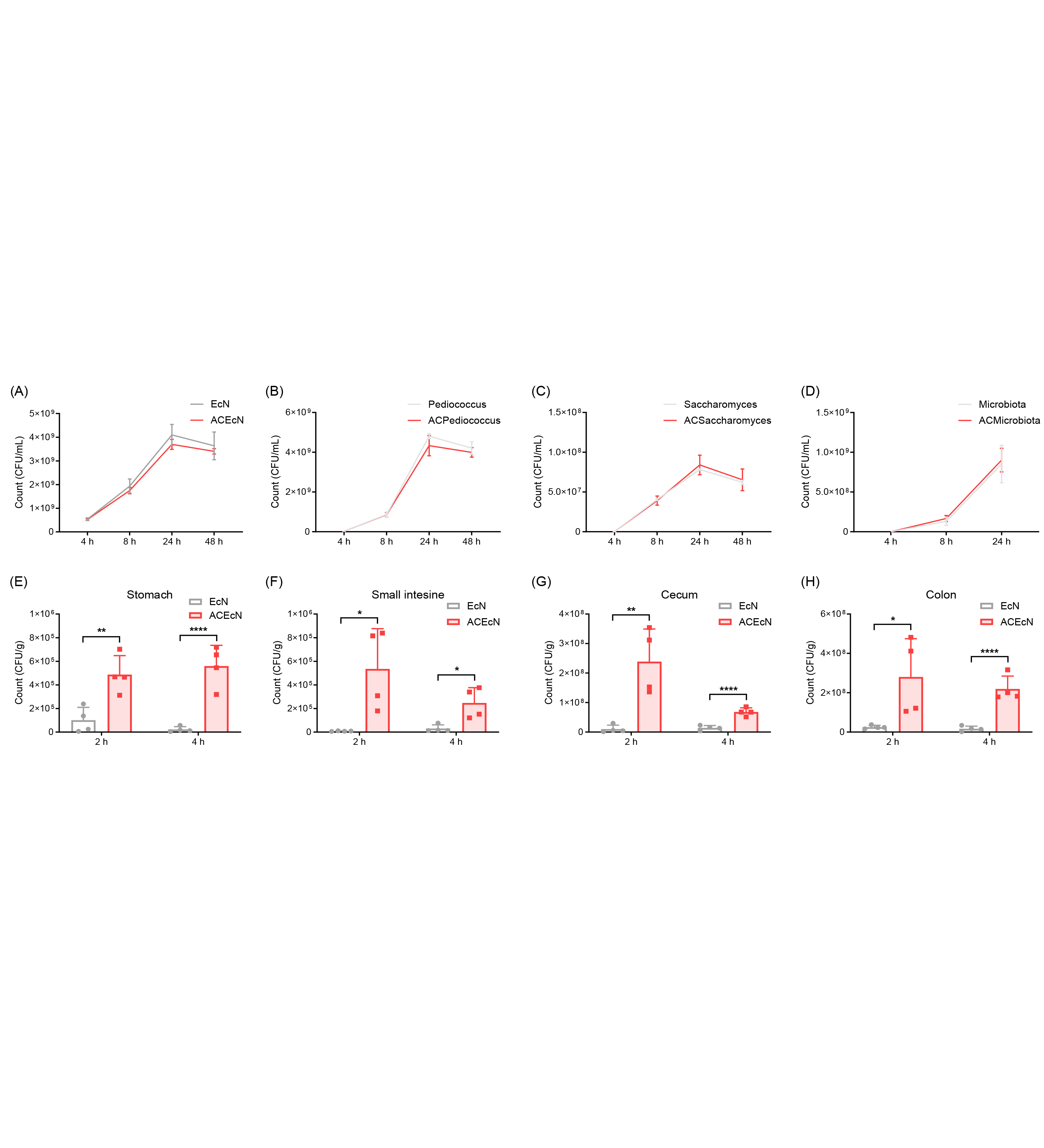


**Figure S12** **Gastrointestinal resistance of encapsulated microbiota.** (A-D) Growth curves for coated EcN, *Pediococcus*, *Saccharomyces*, and microbiota (n = 3). (E-H) EcN colonization quantified by plate counts in the stomach, small intestine, cecum, and colon (n = 4). Each independent experiment was repeated three times. Error bars represent standard error of mean. ^*^*p* < 0.05; ^**^*p* < 0.01; ^****^*p* < 0.0001; unpaired *t*-test (and nonparametric tests).


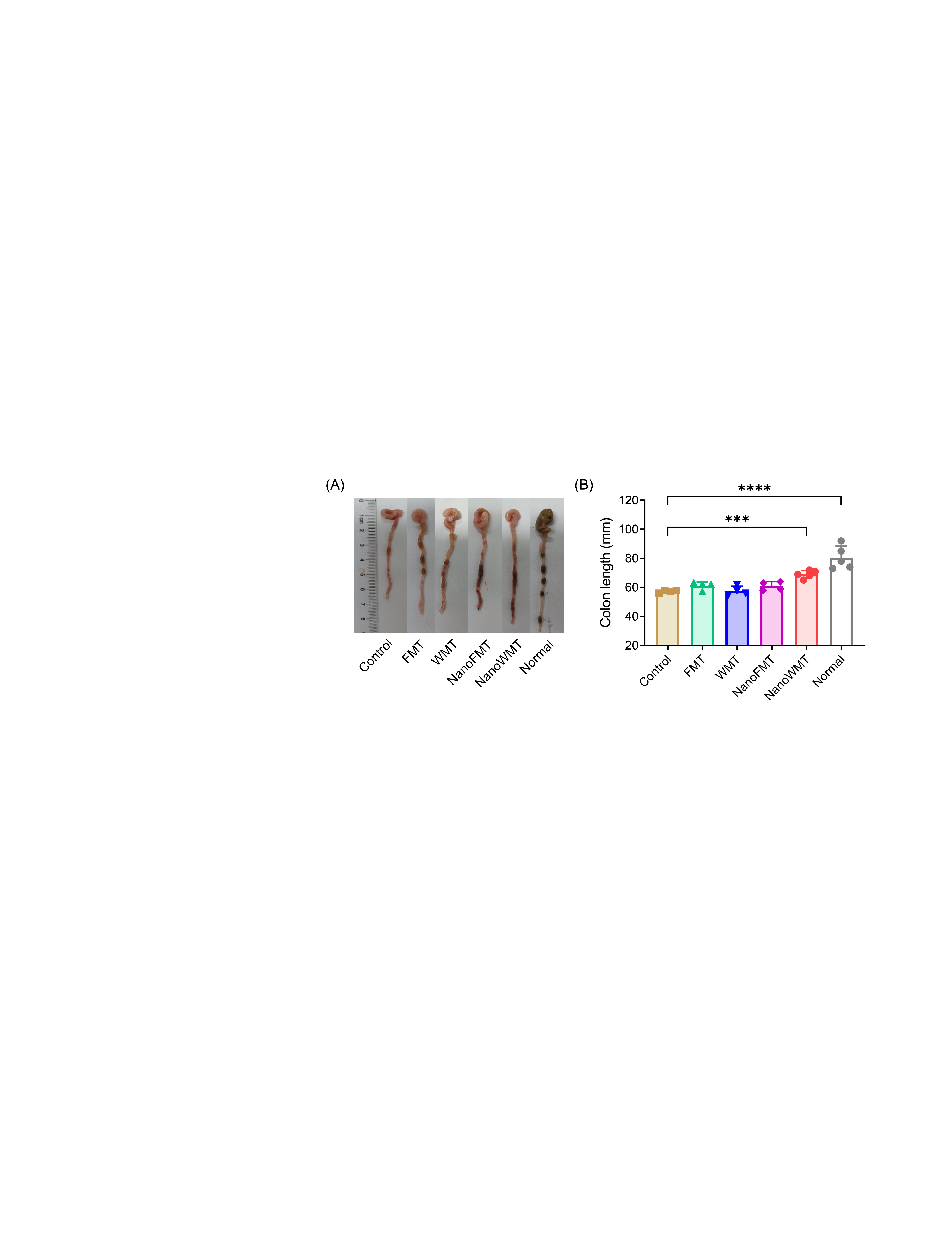


**Figure S13 NanoWMT treatment significantly alleviated the shortening of colon length.** (A) Macroscopic colon images. (B) Colon length quantification (n = 4 or 5). ^***^*p* < 0.001; ^****^*p* < 0.0001; one-way ANOVA (and nonparametric or mixed) with Benjamini-Hochberg correction.

**
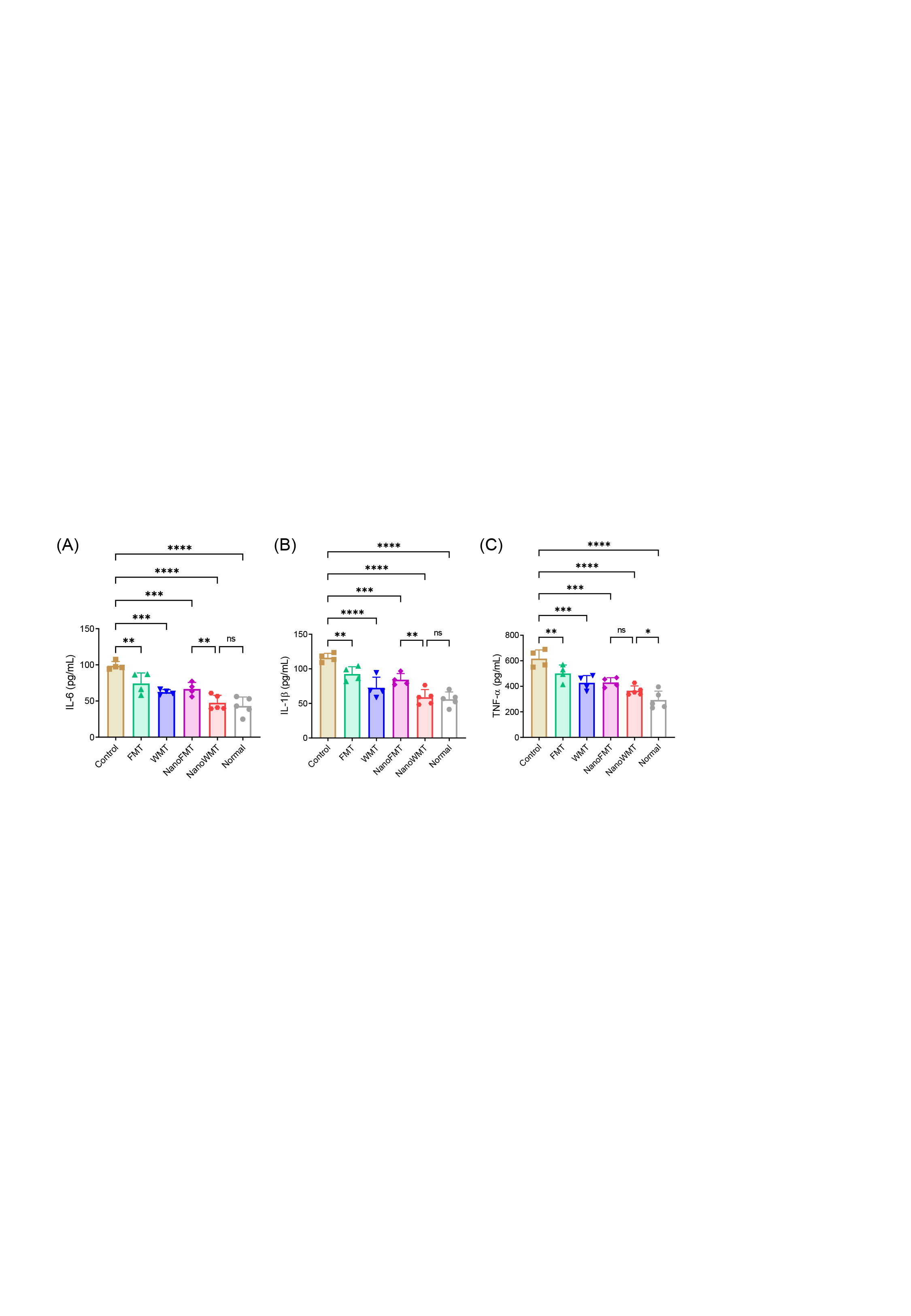
**

**Figure S14** **Serum levels of IL-6, IL-1β, and TNF-α.** Error bars represent standard error of mean (n = 4 or 5). Each independent experiment was repeated three times. ^*^*p* < 0.05; ^**^*p* < 0.01; ^***^*p* < 0.001; ^****^*p* < 0.0001; ns, no significance; one-way ANOVA (and nonparametric or mixed) with Benjamini-Hochberg correction.


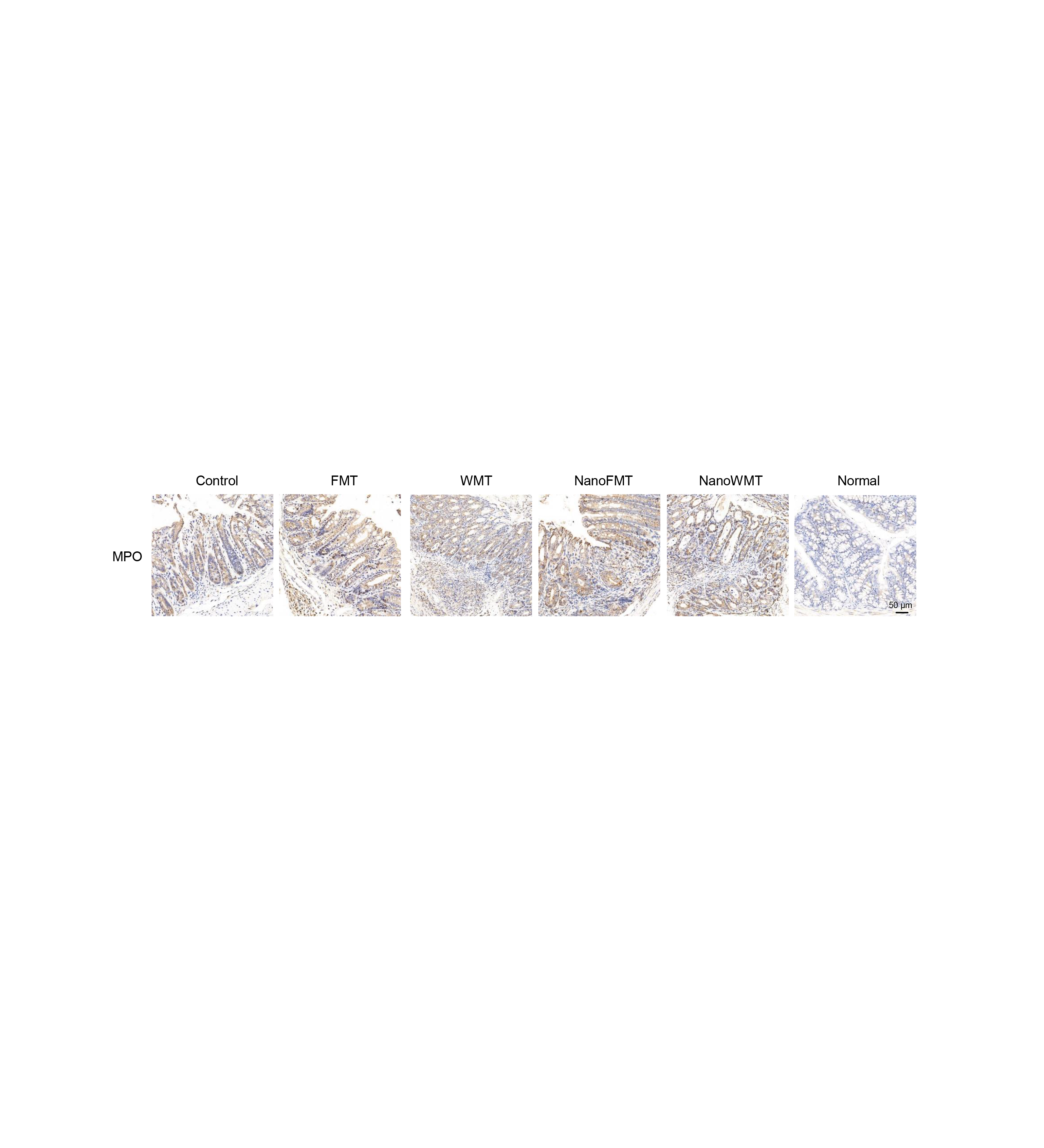


**Figure S15** **MPO immunohistochemistry of distal colon tissue.** Tissue samples were obtained on the sixth day of the experiment (scale bar, 50 μm).


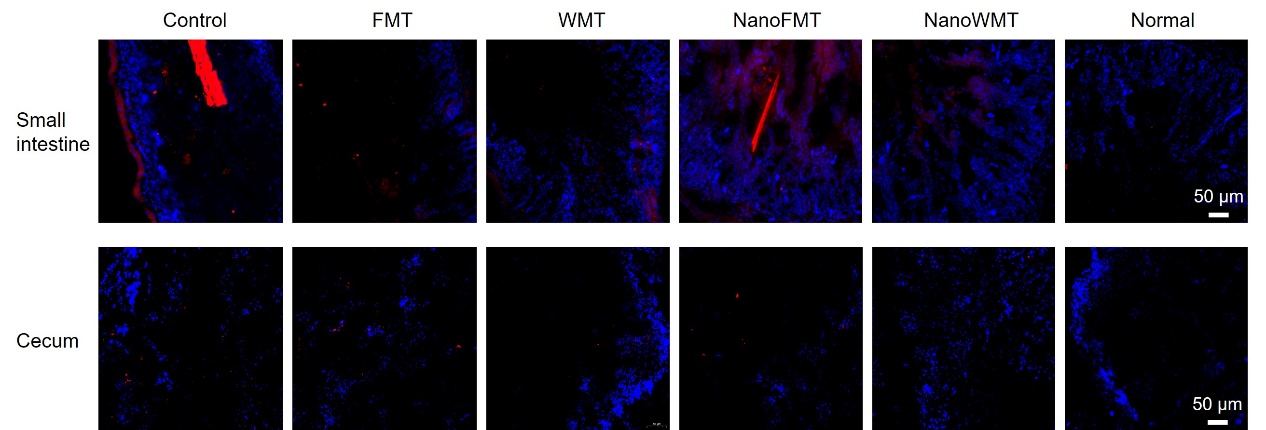


**Figure S16** **Immunofluorescence images of mCherry-labeled STm in the small intestine and cecum.** Tissue samples were obtained on the sixth day of the experiment (scale bar, 50 μm).


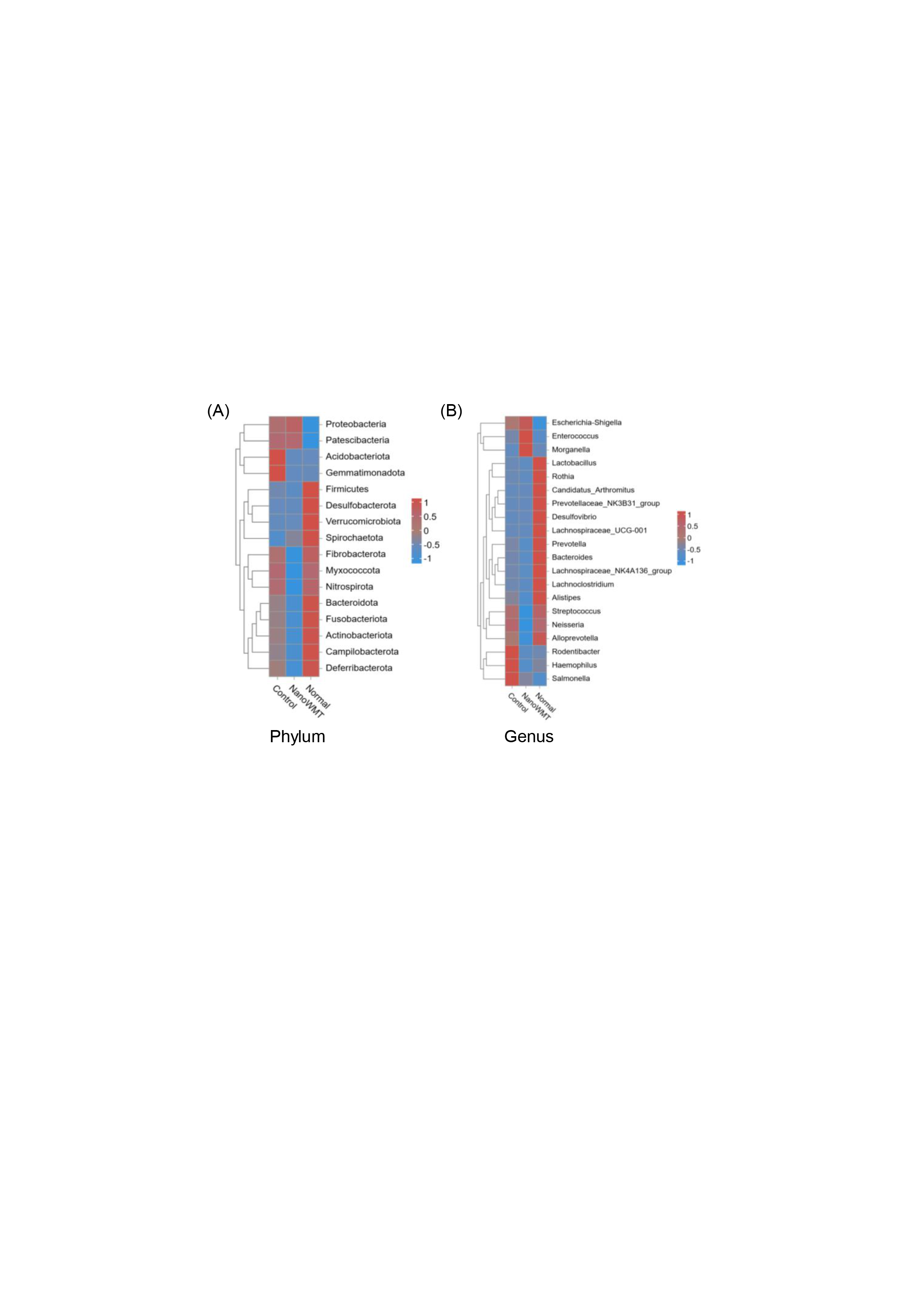


**Figure S17** **NanoWMT reduces the harmful bacteria abundance in the small intestinal contents of STm-induced colitis mice.** Species abundance heatmap at the phylum (A) and genus (B) levels, (n = 4).

**
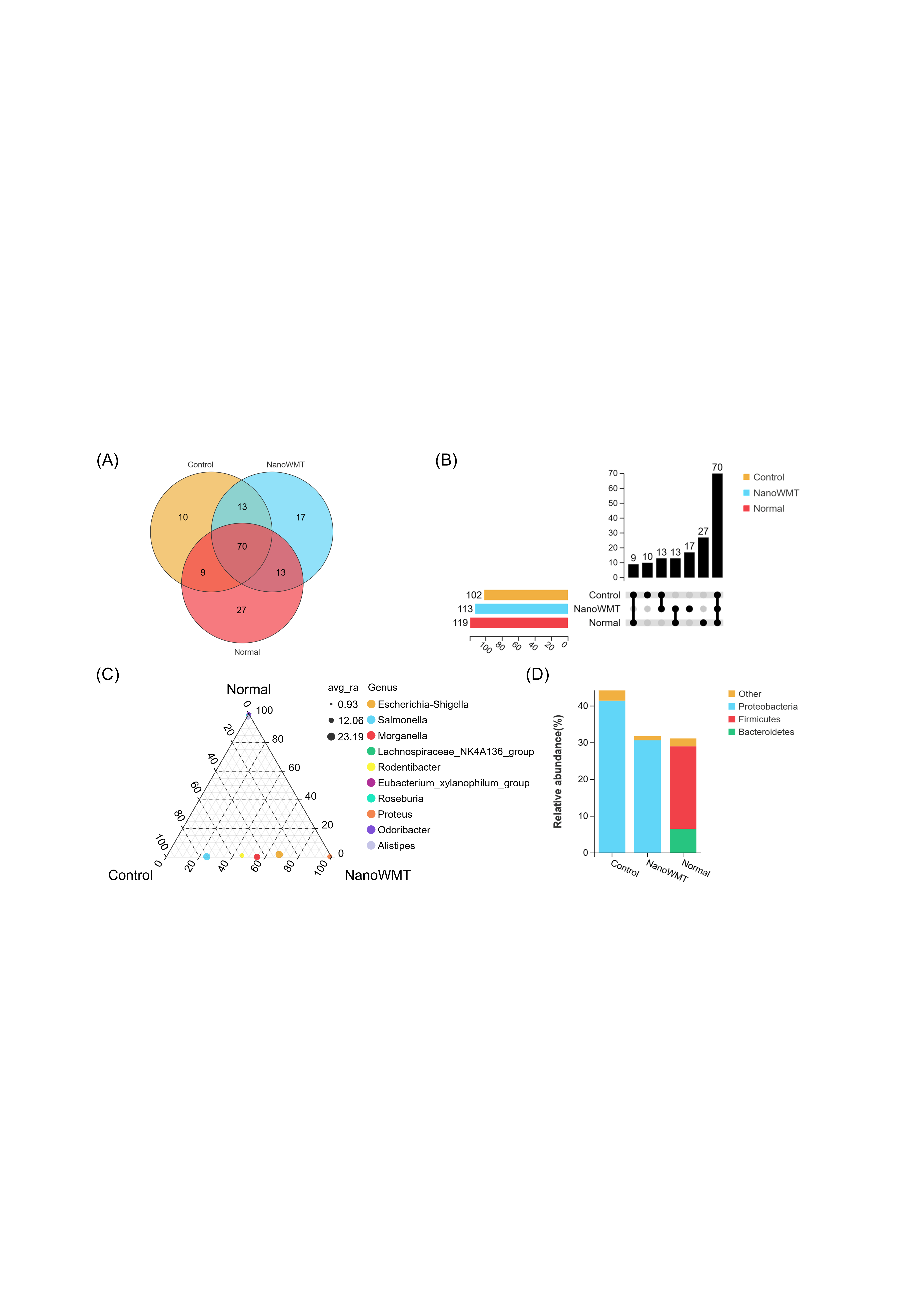
**

**Figure S18** **Bacterial composition and differential abundance of the cecal contents in STm-induced colitis mice.** (n = 4) Venn diagram (A) and Upset plot (B) display the number of species in each group at the genus level. (C) Ternary plot shows species distribution at the genus level. (D) Abundance of biofilm forming bacteria.

**
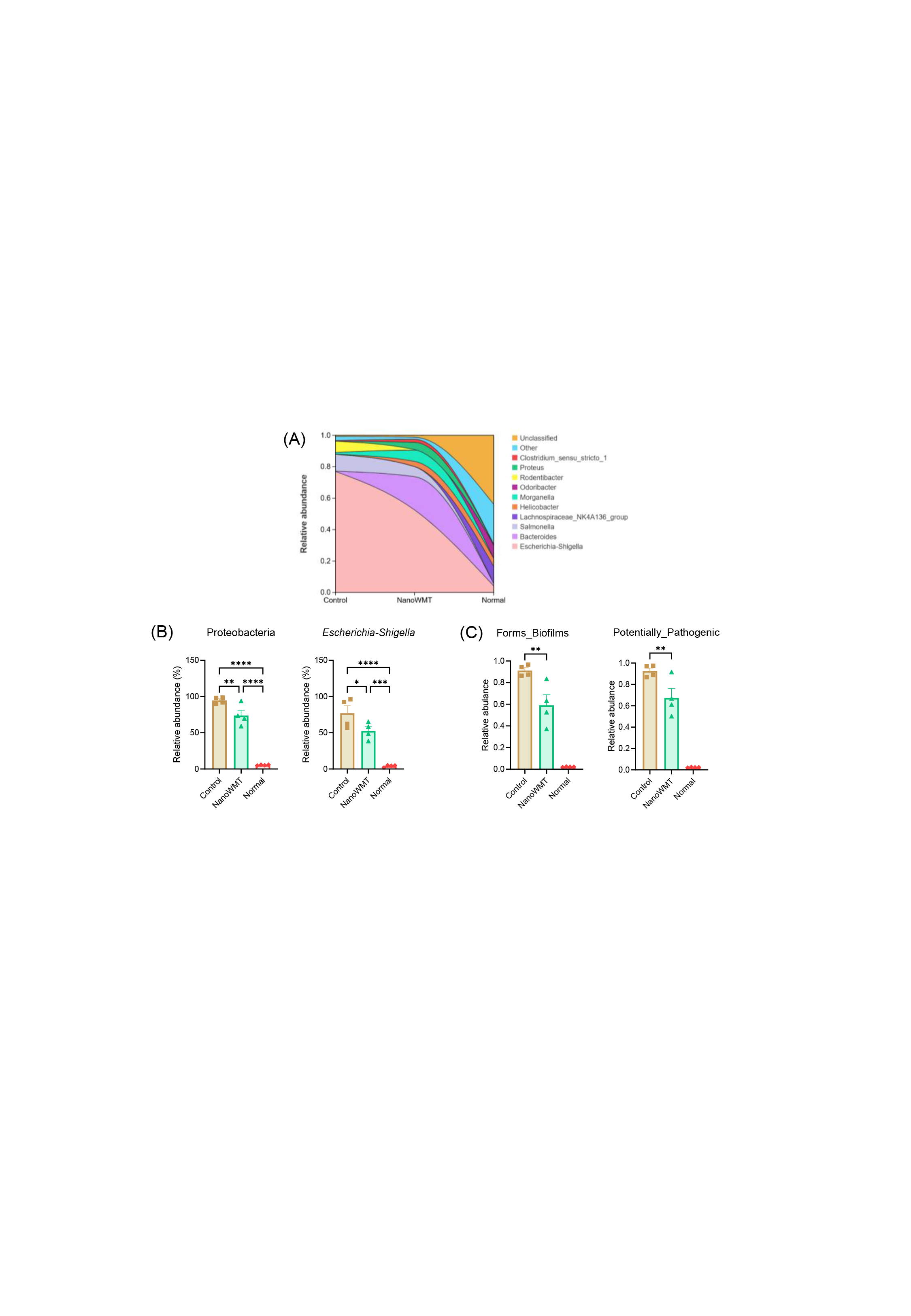
**

**Figure S19** **Bacterial composition and differential abundance of the colonic contents in STm-induced colitis mice.** (A) Species distribution river map between Control, NanoWMT and Normal groups. (B) Relative abundance of representative microbiota in colon. (C) Phenotypic abundance to predict the relative abundance of bacterial phenotypes. Error bars represent standard error of mean (n = 4). ^*^*p* < 0.05; ^**^*p* < 0.01; ^***^*p* < 0.001; ^****^*p* < 0.0001; one-way ANOVA (and nonparametric or mixed) with Benjamini-Hochberg correction.


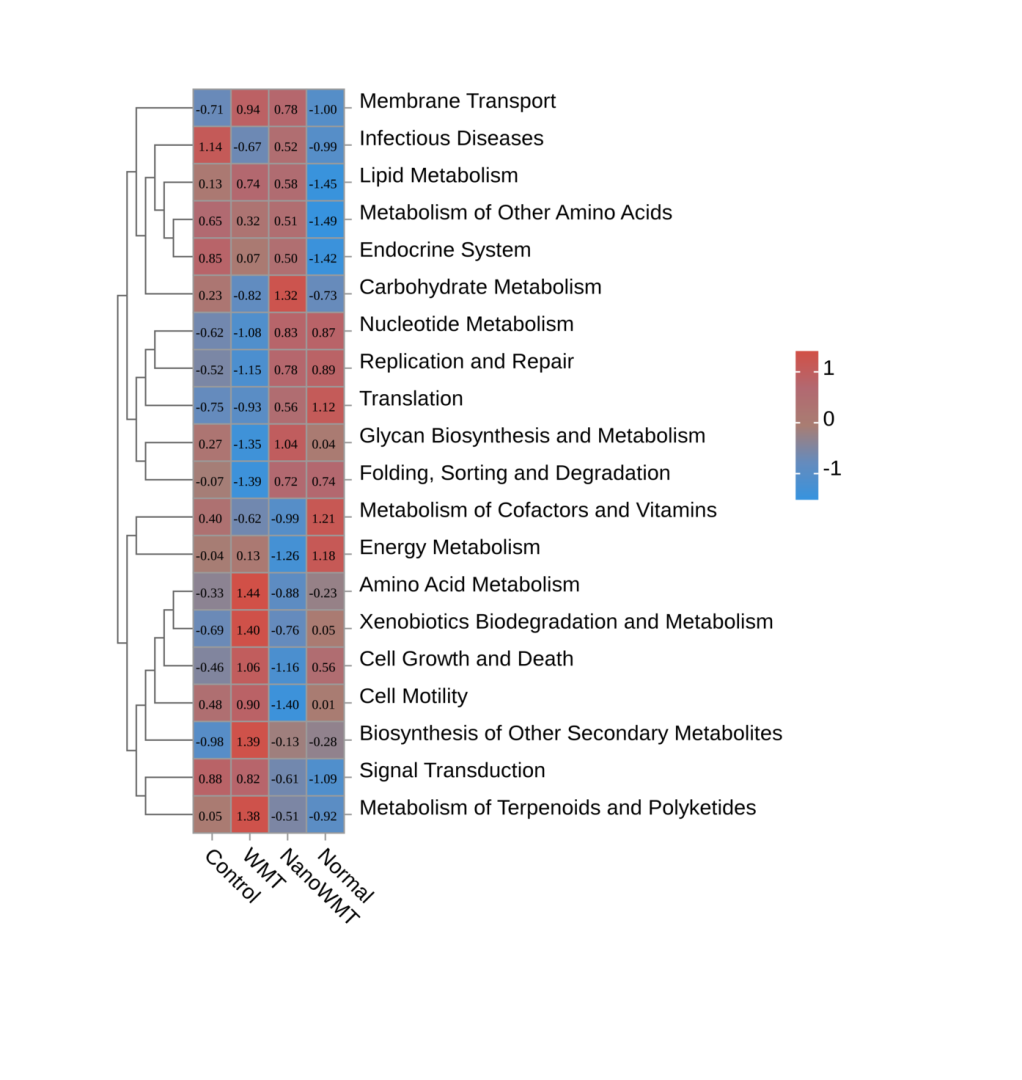


**Figure S20** **Heatmap of functional abundance.** To predict the relative abundance of bacterial functions in the colon of STm-induced colitis mice.


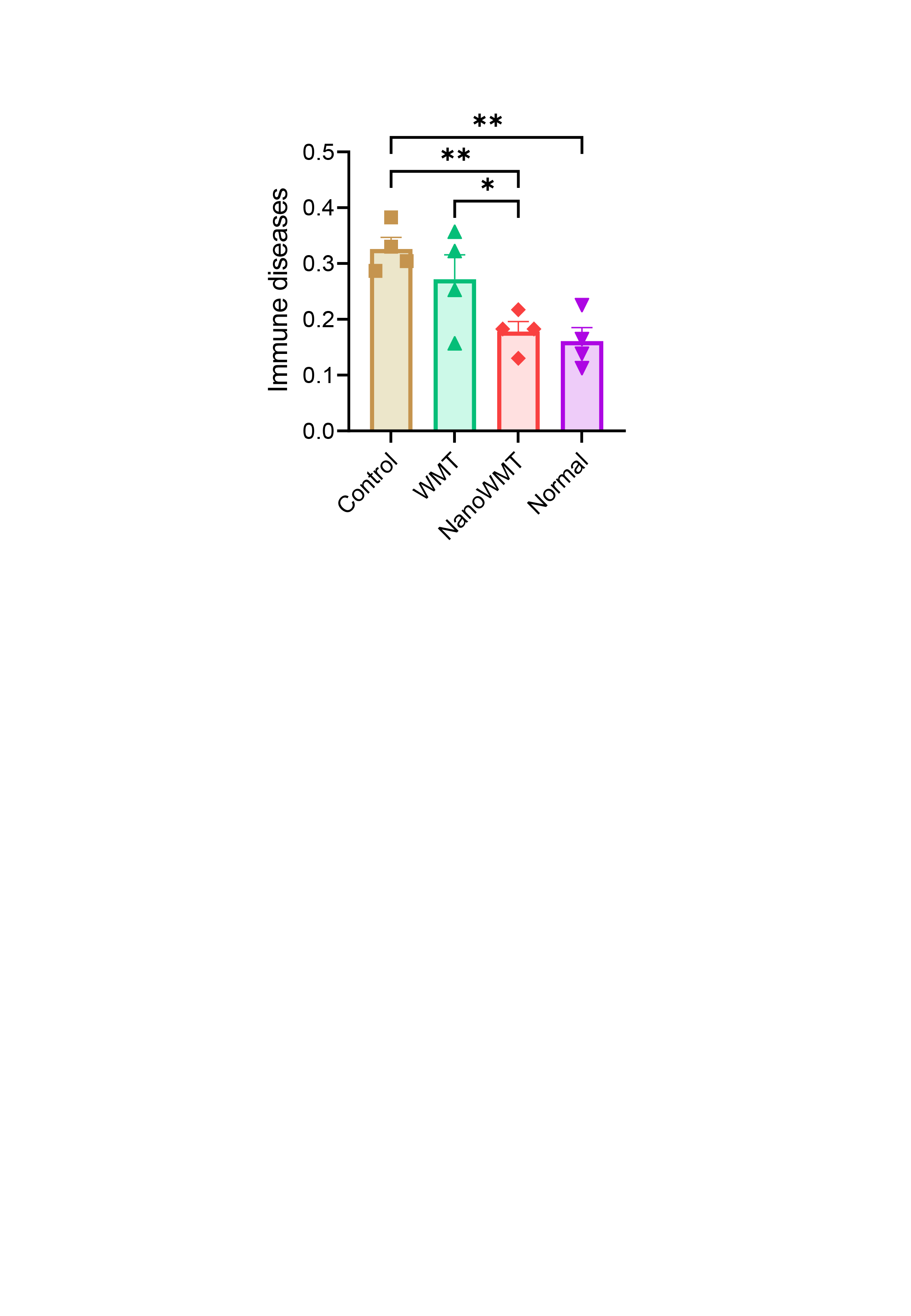


**Figure S21** **Functional prediction analysis of gut microbiota based on PICRUSt in the colon of** **STm-induced colitis mice.** Error bars represent standard error of mean (n = 4). ^*^*p* < 0.05; ^**^*p* < 0.01; one-way ANOVA (and nonparametric or mixed) with Benjamini-Hochberg correction.

**REFERENCES**
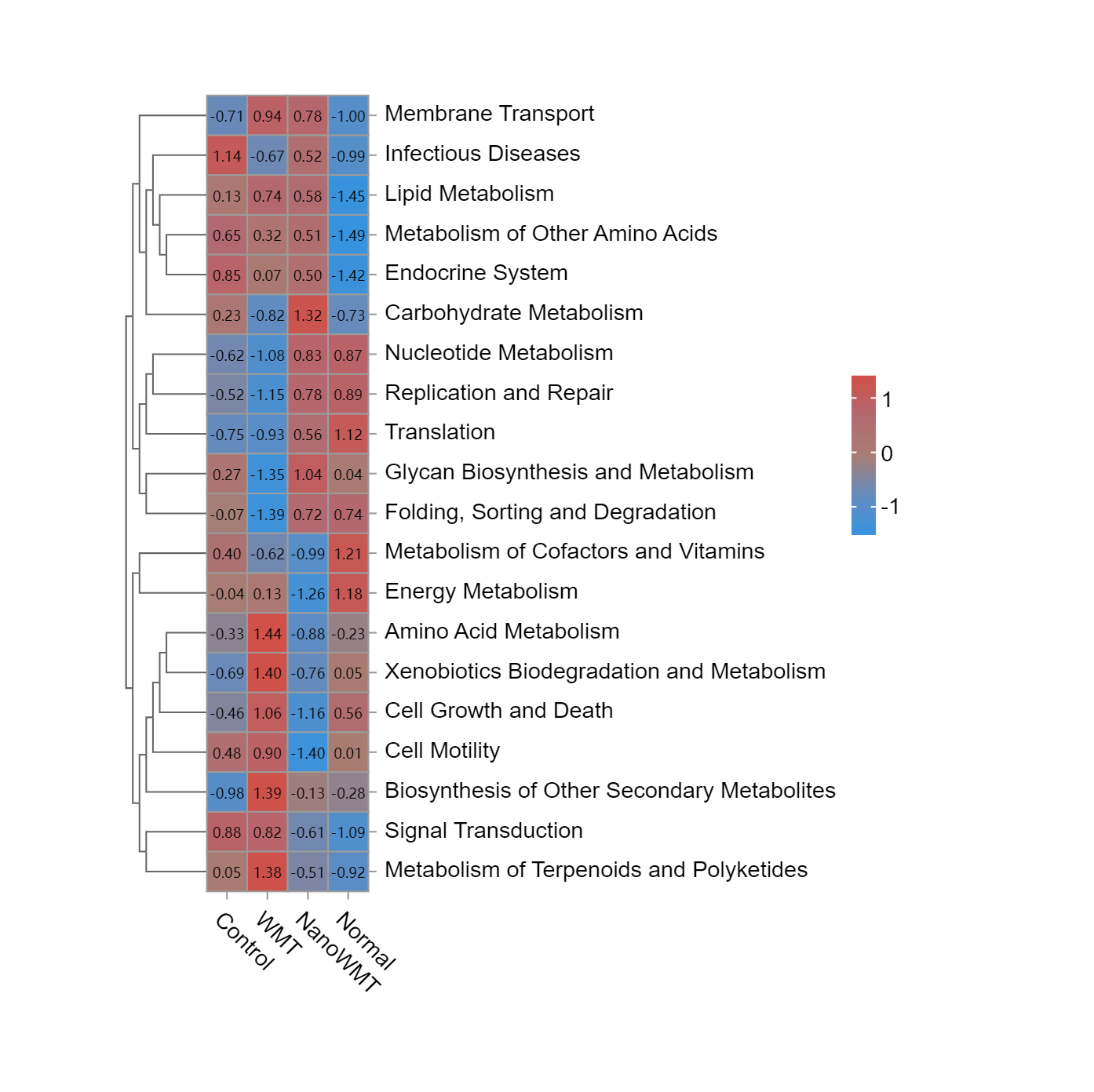


1. Kastl, Arthur J., Natalie A. Terry, Gary D. Wu, Lindsey G. Albenberg. 2020. “The Structure and Function of the Human Small Intestinal Microbiota: Current Understanding and Future Directions.” *Cellular and Molecular Gastroenterology and Hepatology* 9: 33-45. <https://doi.org/10.1016/j.jcmgh.2019.07.006>

2. Hou, Weiliang, Juanjuan Li, Zhenping Cao, Sisi Lin, Chao Pan, Yan Pang, Jinyao Liu. 2021. “Decorating Bacteria with a Therapeutic Nanocoating for Synergistically Enhanced Biotherapy.” *Small* 17: e2101810. <https://doi.org/10.1002/smll.202101810>

3. Hou, Weiliang, Yuan Cao, Jifeng Wang, Fang Yin, Jiahui Wang, Ning Guo, Ziyi Wang, et al. 2025. “Single-cell nanocapsules of gut microbiota facilitate fecal microbiota transplantation.” *Theranostics* 15: 2069-2084. <https://doi.org/10.7150/thno.104852>

4. Barthel, Manja, Siegfried Hapfelmeier, Leticia Quintanilla-Martínez, Marcus Kremer, Manfred Rohde, Michael Hogardt, Klaus Pfeffer, Holger Rüssmann, Wolf-Dietrich Hardt. 2003. “Pretreatment of mice with streptomycin provides a Salmonella enterica serovar Typhimurium colitis model that allows analysis of both pathogen and host.” *Infect Immun* 71: 2839-2858. <https://doi.org/10.1128/iai.71.5.2839-2858.2003>

5. Wang, Xinwu, Yating Xing, Yalu Ji, Hengyu Xi, Xiaohe Liu, Li Yang, Liancheng Lei, Wenyu Han, Jingmin Gu. 2022. “The Combination of Phages and Faecal Microbiota Transplantation Can Effectively Treat Mouse Colitis Caused by Salmonella enterica Serovar Typhimurium.” *Front Microbiol* 13: e944495. <https://doi.org/10.3389/fmicb.2022.944495>
